# Domestic versus foreign drivers of trade (im)balances: How robust is evidence from estimated DSGE models?^☆^

**DOI:** 10.1016/j.jimonfin.2021.102509

**Published:** 2022-03

**Authors:** Roberta Cardani, Stefan Hohberger, Philipp Pfeiffer, Lukas Vogel

**Affiliations:** a European Commission, Joint Research Centre (JRC), Ispra, Italy; b European Commission, Directorate General for Economic and Financial Affairs, Brussels, Belgium

**Keywords:** Agnostic structural disturbances, Open economy DSGE model, Trade balance, Germany, Spain

## Abstract

Estimated DSGE models tend to ascribe a significant and often predominant part of a country's trade balance (TB) dynamics to domestic drivers (“shocks”), suggesting foreign factors to be only of secondary importance. This paper revisits the result based on more agnostic approaches to shock transmission and using “agnostic structural disturbances”. We estimate multi-region models for Germany and Spain as countries with very distinct TB patterns since 1999. Results suggest that domestic drivers remain dominant when theory-based restrictions on shock transmission are relaxed, although the transmission of foreign shocks is strengthened.

## 1 Introduction

The sign and size of a country's trade balance (TB) are affected by domestic and foreign factors alike (e.g., Obstfeld and Rogoff, 1996). The relative importance of each group is an empirical question, which we revisit through the lens of dynamic stochastic general equilibrium (DSGE) models, with a focus on Germany (DE) and Spain (ES) as two large Euro Area (EA) Member States that have witnessed strikingly distinct TB dynamics since the start of EMU in 1999. In particular, we estimate multi-region DSGE models (EA Member State, the rest of the EA (REA), and the rest of the world (RoW)) for DE and ES, respectively.

Estimated DSGE models have been a popular tool for decomposing macroeconomic dynamics into fundamental drivers since the early 2000s. Their appeal lies, on the theoretical side, in the explicit modelling of market interactions in the macroeconomy and, on the empirical side, in the extensive use of available information to identify model parameters (transmission channels) and exogenous shocks (drivers). Estimated open-economy DSGE models tend to attribute fluctuations in economic activity and net trade in advanced economies primarily to domestic factors, however, despite the inclusion of foreign regions and shocks. A classic reference for low spillover is Justiniano and Preston (2010), who show that US shocks account for only 1–3% of output and real effective exchange rate (REER) volatility in an estimated open-economy DSGE model of the Canadian economy (1982–2007). Results in Kollmann et al., 2016, Giovannini et al., 2019 with more complex estimated multi-region models are more balanced, with non-negligible contributions of RoW shocks to EA and US GDP and trade balance dynamics since 1999. Turning to individual EA countries, Kollmann et al. (2015) find the persistence of DE's TB surplus to be driven mainly by domestic factors, although external factors matter quantitatively in the build-up phase. Looking at Spain, in 't Veld et al., 2014, in ’t Veld et al., 2015) find a quantitatively significant contribution of narrowing intra-EA risk premia to the country's TB deficit before the financial crisis, but little contribution of (other) foreign factors to GDP growth and net export dynamics. Albonico et al. (2019) provide a comparative perspective in which domestic shocks are essential to explain the persistent TB surplus of DE and the pre-crisis TB deficit build-up in ES and Italy (IT), whereas foreign shocks account for a more substantial part in France, particularly in recent years.

The finding of a limited role for external factors is linked to the ambivalent role of various shocks in terms of spillover and TB dynamics. Positive foreign *supply* shocks, e.g., tend to generate positive income effects and, at the same time, improve the competitiveness of foreign producers, where the first effect strengthens and the second effect weakens net exports of the domestic economy. Spillover of positive foreign *demand* shocks inside a monetary union is rather weak in *normal times*, as stronger demand is met by a tightening of monetary policy that dampens domestic demand in the domestic economy and appreciates the common currency, reducing net exports to the RoW. In addition, it should be underlined that distinguishing between domestic and foreign shocks and between demand and supply shocks is less clear-cut in reality. Domestic demand shocks, e.g., can be the result of changes in the credit supply by foreign or domestic financial intermediaries, and domestic financial conditions may be subject to contagion effects (e.g., Dornbusch et al., 2000) in excess of “real” trade and financial linkages.

The benchmark estimates in this paper are in line with previous findings and suggest TB dynamics in DE and ES to be driven mainly by domestic (demand) factors. In a counterfactual simulation without domestic demand shocks, Spain's TB (in % of nominal GDP) is more than 5 percentage points (pp) higher in 2008, and the subsequent TB reversal 4 pp less pronounced. Domestic demand conditions also explain a large part of the DE TB variation, but external factors are more important than for ES. The REA pre-crisis boom raised DE net exports, whereas falling REA demand has weighted negatively on the DE TB during the subsequent recession. We find little role for spillover of foreign *supply* shocks (“competitiveness gains/losses”) within the EA, however. Intra-EA relative price dynamics reflect to a large extent diverging demand conditions in the benchmark model.

We then extend the model in two directions to assess the robustness of the benchmark results. We first investigate the hypothesis that price and wage pressure from abroad affects net trade beyond the ambivalent role of supply shocks in the standard model and the (realised) transmission to export and import prices. We test the idea by including price and wage shocks directly in the trade equations and re-estimating the models with wide agnostic priors. The data reject the inclusion of supply shocks in trade equations for Spain, supporting the results of the baseline model. Interestingly, however, we find some evidence for a stronger role of price pressure and spillover in the case of DE, with a larger role for foreign price shocks. The inclusion of foreign price shocks in trade equations does, however, not overturn the benchmark result that domestic demand factors dominate the decomposition of TB dynamics in DE and ES.

Second, we follow recent methodological advances in empirical business cycle analysis and adopt a more agnostic perspective on shock transmission by applying the *agnostic structural disturbances* (ASDs) methodology of Den Haan and Drechsel (2021). ASDs enter the model like structural shocks, but their impact is *a priori* unrestricted. Our main results remain robust with different ASD specifications, i.e. domestic developments still explain most of the TB variance. One of the ASDs that we investigate shares essential similarities with the key domestic demand shock, but also implies stronger international co-movement in the spirit of global risk shocks. We conclude that the (more) agnostic specifications preserve the main message from the benchmark model.

## 2 Stylised facts

**Fig. 1:**
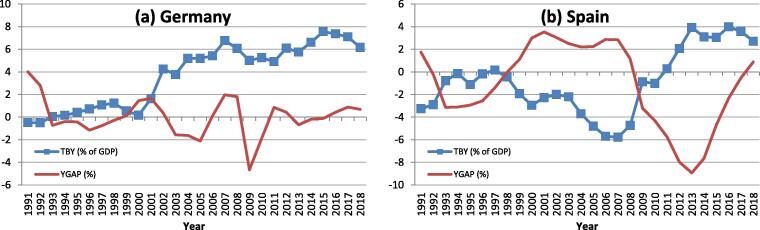
**Trade balance and output gap** Note: TBY is the trade balance in % of GDP; YGAP is the output gap in %. Source: AMECO.

The TBs of DE and ES have followed distinct patterns in recent years (Fig. 1). DE is characterised by a large and persistent TB surplus that has built up since the early 2000 s, with a pause in the years of the global financial crisis and a peak at around 8% of GDP in 2015. ES has run a large trade deficit in the early years of EMU, reaching −6% of GDP on the eve of the financial crisis. The TB has turned into a surplus of up to 4% of GDP in recent years. DE's TB surplus shows limited co-movement with the output gap, i.e. the surplus has remained high in periods of positive and negative output gaps alike. Spain's TB dynamics, to the contrary, displays marked cyclicality, with TB deficits in periods of positive output gaps, and a move into surplus in conjunction with negative output gaps after 2008.

**Fig. 2:**
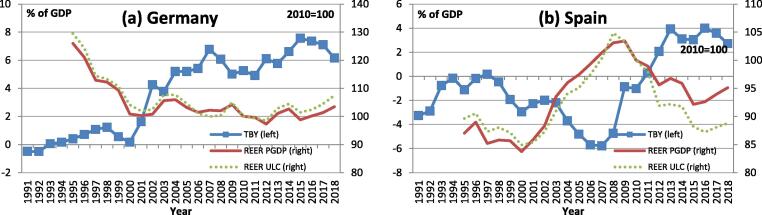
**Trade balance and real effective exchange rate** Note: TBY is the trade balance in % of GDP; REER is the real effective exchange rate, normalised to 2010 = 100. The REER is calculated based on the GDP deflator (PGDP) and unit labour costs (UCL), respectively, compared to a group of 37 industrial countries. A REER decline indicates REER depreciation. Source: AMECO.

Fig. 2 illustrates DE-ES differences with respect to the co-movement between TB and indicators of price competitiveness, namely the REERs based on the GDP deflator (PGDP) and unit labour costs (ULC), respectively. The sustained increase in DE's TB surplus did not coincide with the steady depreciation of DE's REER. The TB of ES, by contrast, co-moved with the REER. The REER appreciated during the pre-crisis boom, when the ES TB moved into deficit, and it depreciated after 2008, when the economy contracted and the TB moved into positive territory.

## 3 Model description

Our analysis uses a set of estimated multi-country models.^1^ The models are ex-ante identical, i.e. they share the same structure and the same set of observed variables used for the estimation. Each model consists of an EA Member State (MS), the REA, and the RoW as building blocks (with country index k). International trade and financial markets link the three regional blocks. The EA MS block is more detailed than the other regions. It assumes two (representative) households, firms and a government. The MS households provide labour services to domestic firms. Final good firms combine domestic and imported intermediate inputs. Intermediates are produced by monopolistically competitive firms using local labour and capital as inputs. Fiscal and monetary authorities follow estimated policy rules.

### 3.1 EA Member State households

The household sector consists of a continuum of households $j\in \left[0;1\right]$. There are two types of households, savers (“Ricardians”, superscript *s*) who own firms and hold government and foreign bonds, and liquidity-constrained households (superscript *c*) who only receive labour and transfer income and do not save. The share of savers in the population is $\omega^{s}$.

Both households enjoy utility from consumption $C_{\mathrm{jkt}}^{r}$ and incur disutility from labour $N_{\mathrm{jkt}}^{r}$ (r=s,c). Ricardian’s utility also depends on the financial assets held. Expected life-time utility of household *r* is defined as:

$$U_{\mathrm{jk}0}^{r}=E_{0}\sum_{t=0}^{\infty }\beta^{t}\Theta_{t}u_{\mathrm{jk}t}^{r}\left(∙\right),\;with\;\Theta_{t}\equiv exp\sum_{s=0}^{t-1}\varepsilon_{s}^{c}$$

where β is the (non-stochastic) discount factor (common for both types of households) and $\varepsilon_{ks}^{c}$ is a saving shock, which is limited to saver households.^2^ $u_{\mathrm{jkt}}^{r}\left(∙\right)$ denotes per-period utiliy described below.

#### 3.1.1 Ricardian households

The Ricardian households work, consume, own firms and receive nominal transfers $T_{\mathrm{jkt}}^{s}$ from the government. Ricardians are the only households with full access to financial markets. The financial wealth of household *j* consists of bonds and shares, where $P_{\mathrm{kt}}^{S}$ is the nominal price of shares in *t*, $B_{\mathrm{jkt}-1}^{S}$ the number of shares held by the household, and $P_{\mathrm{kt}}^{C,vat}$ is the consumption price, including VAT. The period *t* budget constraint of a saver household *j* is:

$$\left(1-\tau_{k}^{N}\right)W_{\mathrm{kt}}N_{\mathrm{jkt}}^{s}+\left(1+i_{\mathrm{kt}-1}^{g}\right)B_{\mathrm{jkt}-1}^{g}+\left(1+i_{t-1}^{\mathrm{bw}}\right)e_{\mathrm{RoW},EA,t}B_{\mathrm{jkt}-1}^{\mathrm{bw}}+\left(1+i_{kt-1}^{\mathrm{rf}}\right)B_{\mathrm{jkt}-1}^{\mathrm{rf}}+\left(P_{\mathrm{kt}}^{S}+P_{\mathrm{kt}}^{Y}d_{\mathrm{kt}}\right)B_{\mathrm{jkt}-1}^{S}+T_{kt}-tax_{kt}=P_{\mathrm{kt}}^{C,vat}C_{\mathrm{jkt}}^{s}+A_{\mathrm{jkt}}+adj_{kt}^{W},$$

where $W_{\mathrm{kt}}$ is the nominal wage rate, $N_{\mathrm{jkt}}^{s}$ is the employment in hours, and $\tau_{k}^{N}$ the labour tax rate. $B_{\mathrm{jkt}-1}^{g}$, $B_{\mathrm{jkt}-1}^{bw}$ and $B_{\mathrm{jkt}-1}^{rf}$ are domestic government bonds, foreign bonds, and risk-free private bonds with returns $i_{\mathrm{kt}-1}^{g},$ $i_{t-1}^{\mathrm{bw}}$, and $i_{kt-1}^{\mathrm{rf}}$, respectively.^3^ $P_{\mathrm{kt}}^{Y}$ is the GDP price deflator. $e_{RoW,kt}$ denotes the bilateral exchange rate towards the RoW. $T_{kt}$ are government transfers to savers, and $tax_{kt}$ are lump-sum taxes paid by savers. Intermediate goods producers pay dividends ${P_{\mathrm{kt}}^{Y}d}_{\mathrm{kt}}$ to savers. $\mathrm{ad}j_{kt}^{W}$denotes wage adjustment costs.

We define the gross nominal return on domestic shares as:

$$1+i_{\mathrm{kt}}^{s}=\frac{P_{\mathrm{kt}}^{S}+P_{\mathrm{kt}}^{Y}d_{\mathrm{kt}}^{\;}}{P_{\mathrm{kt}-1}^{S}}$$

The instantaneous utility functions of savers, $u^{s}\left(∙\right)$, is defined as:

$$u_{\mathrm{jkt}}^{s}\left(C_{\mathrm{jkt}}^{s},N_{\mathrm{jkt}}^{s},\frac{U_{\mathrm{jkt}-1}^{A}}{P_{\mathrm{kt}}^{c,vat}}\right)=\frac{1}{1-\theta_{k}}{\left(C_{\mathrm{jkt}}^{s}-h_{k}C_{\mathrm{kt}-1}^{s}\right)}^{1-\theta_{k}}-\frac{\omega_{k}^{N}\varepsilon_{kt}^{U}}{1+\theta_{k}^{N}}{\left(C_{kt}^{\;}\right)}^{1-\theta_{k}}{\left(N_{jkt}^{s}\right)}^{1+\theta_{k}^{N}}-{{\bar{\lambda }}^{s}}_{kt}\frac{U_{jkt-1}^{A}}{P_{kt}^{c,vat}}$$

where $C_{\mathrm{kt}}^{s}=\int_{0}^{\omega^{s}}C_{\mathrm{jkt}}^{s}dj$, and $C_{\mathrm{kt}}=\omega^{s}C_{\mathrm{kt}}^{s}+{\left(1-\omega^{s}\right)C}_{\mathrm{kt}}^{c}$, $h_{k}\in \left(0,1\right)$ measures the strength of external habits in consumption, $\omega_{k}^{N}$ the weight of the disutility of labour, and $\varepsilon_{\mathrm{kt}}^{U}$ captures a labour supply (or wage mark-up) shock.

The disutility of holding risky financial assets, $U_{\mathrm{jkt}-1}^{A},$ is defined as:

$$U_{\mathrm{jkt}-1}^{A}=\left(\alpha_{k}^{b0}+\varepsilon_{kt-1}^{B}\right)B_{jkt-1}^{G}+\left(\alpha_{k}^{bw0}+\varepsilon_{kt-1}^{bw}\right)e_{RoW,\mathrm{EA},t-1}B_{jkt-1}^{W}+\frac{\alpha_{k}^{bw1}}{2}\frac{{\left(e_{RoW,\mathrm{EA},t-1},B_{kt-1}^{W}\right)}^{2}}{P_{kt-1}^{Y}Y_{kt-1}}+\left(\alpha_{k}^{s0}+\varepsilon_{kt-1}^{S}\right)P_{kt-1}^{S}S_{jkt-1}.$$

The asset-specific risk premium shock depends on an asset-specific exogenous shock $\varepsilon_{t}^{Q},Q\in \left\{g,S,bw\right\}$ (government bonds, stocks, and foreign assets) and an asset-specific intercept $\alpha^{x},\;x\in \{b0,s0,bw0\}$.^4^ Similar to (Fisher, 2015, Krishnamurthy and Vissing-Jorgensen, 2012), introducing a disutility of holding risky assets captures the households’ preferences for the safe short-term bonds and introduces an endogenous wedge between the return on risky assets and safe bonds.

An uncovered interest rate parity condition links the interest rate of the MS to the EA interest rate:

$$i_{kt}^{rf}=i_{EA,t}-\left(\alpha_{k}^{bw1}\frac{e_{\mathrm{RoW},EA,t}B_{\mathrm{kt}}^{bw}}{P_{\mathrm{kt}}^{Y}Y_{\mathrm{kt}}}\right)+\varepsilon_{\mathrm{kt}}^{\mathrm{FQ}}$$

where $\alpha_{k}^{bw1}\frac{e_{\mathrm{RoW},EA,t}B_{\mathrm{kt}}^{bw}}{P_{\mathrm{kt}}^{Y}Y_{\mathrm{kt}}}$ captures a debt-dependent country risk premium on net foreign asset (NFA) holdings to ensure the long-run stability of foreign debt (see, e.g., Schmitt-Grohe and Uribe, 2003). The ‘flight-to-safety’ shock, $\varepsilon_{\mathrm{kt}}^{\mathrm{FQ}}$, creates a wedge between the EA interest rate, $i_{\mathrm{EAt}}$, and $i_{\mathrm{kt}}^{\mathrm{rf}}$. A positive shock increases the required return on domestic assets and the cost of capital, reducing consumption and investment simultaneously. Appendix B.3 provides additional information on the transmission and the relative importance of domestic demand shocks.

#### 3.1.2 Liquidity-constrained household

The liquidity-constrained household consumes her disposable after-tax wage and transfer income in each period (“hand-to-mouth”), which gives the period *t* budget constraint:

$$\left(1+\tau_{\;}^{C}\right)P_{\mathrm{kt}}^{C}C_{\mathrm{jkt}}^{c}=\left(1-\tau_{k}^{N}\right)W_{\mathrm{kt}}N_{\mathrm{jkt}}^{c}+T_{\mathrm{kt}}^{}-{\mathrm{tax}}_{\mathrm{kt}}.$$

#### 3.1.3 Wage setting

Households provide differentiated labour services, $N_{\mathrm{jkt}}^{r}$, in a monopolistically competitive market. A labour union bundles labour hours provided by both types of domestic households and resells homogeneous labour services to intermediate goods producing firms.^5^ The resulting wage rule equates a weighted average of the marginal utility of leisure to a weighted average of the marginal utility of consumption times the real wage adjusted for a wage mark-up. Wage adjustment costs give rise to nominal wage rigidity. We also allow for real wage rigidity as in Blanchard and Galí (2007), parametrised by $\gamma_{k}^{\mathrm{wr}}$.

### 3.2 EA Member State production sector

Perfectly competitive firms produce total output, $O_{\mathrm{kt}}$, by combining value added, $Y_{\mathrm{kt}}$, with energy input, ${\mathrm{Oil}}_{\mathrm{kt}}$, using the following CES production function:

$$O_{\mathrm{kt}}={\left[{\left(1-s_{k}^{\mathrm{Oil}}\right)}^{\frac{1}{\sigma_{k}^{o}}}{\left(Y_{\mathrm{kt}}\right)}^{\frac{\sigma_{k}^{o}-1}{\sigma_{k}^{o}}}+{\left(s_{k}^{\mathrm{Oil}}\right)}^{\frac{1}{\sigma_{k}^{o}}}{\left({\mathrm{Oil}}_{\mathrm{kt}}\right)}^{\frac{\sigma_{k}^{o}-1}{\sigma_{k}^{o}}}\right]}^{\frac{\sigma_{k}^{o}}{\sigma_{k}^{o}-1}},$$

where $s_{k}^{\mathrm{Oil}}$ is the energy input share in total output and $\sigma_{k}^{o}$ the elasticity of substitution between the two components.

Domestic final good firms assemble different intermediate varieties into a homogenous good and sell it to domestic final demand packers and exporters (see below 3.3.2). Demand for individual intermediate goods $i\in \left[0;1\right]$ is downward-sloping and follows $Y_{\mathrm{ikt}}={\left(\frac{P_{\mathrm{ikt}}^{Y}}{P_{\mathrm{kt}}^{Y}}\right)}^{-\sigma^{y}}Y_{kt}$. Each variety i is produced by a single firm using total capital, $K_{\mathrm{ikt}-1}^{\mathrm{tot}}$, and labour, $N_{\mathrm{ikt}}^{\;}$, which are combined by a Cobb-Douglas production function:

$$Y_{\mathrm{ikt}}={\left[A_{\mathrm{kt}}^{Y}\left(N_{\mathrm{ikt}}-{\mathrm{FN}}_{\mathrm{ik}}\right)\right]}^{\alpha }{\left({\mathrm{cu}}_{\mathrm{ikt}}K_{\mathrm{ikt}-1}^{\mathrm{tot}}\right)}^{1-\alpha }-A_{\mathrm{kt}}^{Y}{\mathrm{FC}}_{k},$$

where α is the steady-state labour share, $A_{\mathrm{kt}}^{Y}$ is exogenous labour-augmenting productivity common to all firms i.^6^ ${\mathrm{cu}}_{\mathrm{ikt}}$ and ${\mathrm{FN}}_{\mathrm{ik}}$ are the firm-specific level of capital utilisation and labour hoarding, respectively.^7^ Total capital, $K_{\mathrm{ikt}}^{\mathrm{tot}}$, is the sum of private installed capital, $K_{\mathrm{ikt}}$, and public capital, $K_{\mathrm{kt}}^{G}$. ${\mathrm{FC}}_{k}$ captures fixed costs in production.

The monopolistically competitive producers maximize the real value of the firm, $V_{i\mathrm{kt}}$, equal to a discounted stream of future dividends $d_{i\mathrm{kt}}$, $V_{i\mathrm{kt}}=d_{i\mathrm{kt}}+E_{t}\left[{\mathrm{sdf}}_{\mathrm{kt}+1}V_{i\mathrm{kt}+1}\right]$, with the stochastic discount factor:

$${\mathrm{sdf}}_{\mathrm{kt}}\equiv E_{t}\left[\frac{1+i_{kt+1}^{s}}{1+\pi_{kt+1}^{y}}\right]\approx E_{t}\left[\frac{1+i_{kt}^{rf}+rprem_{kt}^{s}}{1+\pi_{kt+1}^{y}}\right],$$

which depends directly on the investment risk premium, ${\mathrm{rprem}}_{\mathrm{kt}}^{s}$. The dividends are defined as:

$$d_{\mathrm{ikt}}=\left(1-\tau_{\;}^{K}\right)\left(\frac{P_{\mathrm{ikt}}^{Y}}{P_{\mathrm{kt}}^{Y}}Y_{\mathrm{ikt}}-\frac{W_{\mathrm{kt}}}{P_{\mathrm{kt}}^{Y}}N_{\mathrm{ikt}}\right)+\tau_{\;}^{K}\delta_{k}{\frac{P_{\mathrm{kt}}^{I}}{P_{\mathrm{kt}}^{Y}}K}_{\mathrm{ikt-1}}-\frac{P_{\mathrm{kt}}^{I}}{P_{\mathrm{kt}}^{Y}}I_{\mathrm{ikt}}-{\mathrm{adj}}_{\mathrm{ikt}},$$

where $I_{\mathrm{ikt}}$ is physical investment, $P_{\mathrm{kt}}^{I}$ is the investment price, $\tau_{\;}^{K}$ is the corporate tax, and $\delta_{k}$ is the capital depreciation rate. ${\mathrm{adj}}_{\mathrm{ikt}}$ summarizes adjustment costs on production factors, namely capital and labour, on capacity utilisation, labour hoarding and investment, and on prices.

### 3.3 Trade

#### 3.3.1 Import sector

The EA MS final aggregate demand component goods, $C_{\mathrm{kt}}$ (private consumption good), $I_{\mathrm{kt}}$ (private investment good), $G_{\mathrm{kt}}$ (government consumption good), and $I_{\mathrm{kt}}^{G}$ (government investment good), as well as $X_{\mathrm{kt}}$ (export good) are produced by perfectly competitive firms by combining domestic output, $O_{\mathrm{kt}}^{Z}$, with imported goods, $M_{\mathrm{kt}}^{Z}$, where $Z=\{C,I,G,I^{G},X\}$, using the following CES technology:

$$Z_{\mathrm{kt}}=A_{\mathrm{kt}}^{p^{z}}{\left[{\left(1-\varepsilon_{\mathrm{kt}}^{M}s_{k}^{M,Z}\right)}^{\frac{1}{\sigma_{k}^{z}}}{\left(O_{\mathrm{kt}}^{Z}\right)}^{\frac{\sigma_{k}^{z}-1}{\sigma_{k}^{z}}}+{\left(\varepsilon_{\mathrm{kt}}^{M}s_{k}^{M,Z}\right)}^{\frac{1}{\sigma_{k}^{z}}}{\left(M_{\mathrm{kt}}^{Z}\right)}^{\frac{\sigma_{k}^{z}-1}{\sigma_{k}^{z}}}\right]}^{\frac{\sigma_{k}^{z}}{\sigma_{k}^{z}-1}},$$

where $A_{\mathrm{kt}}^{p^{z}}$ is a shock to productivity in the sector producing goods, $\varepsilon_{\mathrm{kt}}^{M}$ is a shock to the share of good-specific import demand components, $s_{k}^{M,Z}$, and $\sigma_{k}^{z}$ is the elasticity of substitution between domestic output and imports. It follows that the demand for $O_{\mathrm{kt}}^{Z}$ and imported goods $M_{\mathrm{kt}}^{Z}$ are given by:

$$O_{\mathrm{kt}}^{Z}={\left(A_{\mathrm{kt}}^{p^{z}}\right)}^{\sigma^{z}-1}\left(1-\varepsilon_{\mathrm{kt}}^{M}s_{k}^{M,Z}\right){\left(\frac{P_{\mathrm{kt}}^{O}}{P_{\mathrm{kt}}^{Z}}\right)}^{-\sigma_{k}^{z}}Z_{kt},$$

$$M_{\mathrm{kt}}^{Z}={\left(A_{\mathrm{kt}}^{p^{z}}\right)}^{\sigma^{z}-1}\varepsilon_{\mathrm{kt}}^{M}s_{k}^{M,Z}{\left(\frac{P_{\mathrm{kt}}^{M}}{P_{\mathrm{kt}}^{Z}}\right)}^{-\sigma_{k}^{z}}Z_{kt},$$

where $P_{\mathrm{kt}}^{O}$ and $P_{\mathrm{kt}}^{M}$ are the price deflators associated with $O_{\mathrm{kt}}^{Z}$ and $M_{\mathrm{kt}}^{Z}$, respectively, and the total final good deflator $P_{\mathrm{kt}}^{Z}$ is:

$$P_{\mathrm{kt}}^{Z}={\left(A_{\mathrm{kt}}^{p^{z}}\right)}^{-1}{\left[\left(1-\varepsilon_{\mathrm{kt}}^{M}s_{k}^{M,Z}\right){\left(P_{\mathrm{kt}}^{O}\right)}^{1-\sigma_{k}^{z}}+\varepsilon_{\mathrm{kt}}^{M}s_{k}^{M,Z}{\left(P_{\mathrm{kt}}^{M}\right)}^{1-\sigma_{k}^{z}}\right]}^{\frac{1}{1-\sigma_{k}^{z}}}$$

Perfectly competitive firms produce final imported goods, $M_{\mathrm{kt}}$, by combining country-specific final import goods, $M_{\mathrm{lkt}}$, using a CES production function:

$$M_{\mathrm{kt}}={\left[\sum_{l}{\left(s_{\mathrm{lk}t}^{M}\right)}^{\frac{1}{\sigma_{k}^{\mathrm{FM}}}}{\left(M_{\mathrm{lkt}}\frac{{\mathrm{size}}_{l}}{{\mathrm{size}}_{k}}\right)}^{\frac{\sigma_{k}^{\mathrm{FM}}-1}{\sigma_{k}^{\mathrm{FM}}}}\right]}^{\frac{\sigma_{k}^{\mathrm{FM}}}{\sigma_{k}^{\mathrm{FM}}-1}},$$

where $\sigma_{k}^{\mathrm{FM}}$ is the price elasticity of demand for country l’s goods and $\mathrm{siz}e_{l}$ denotes the share of country l in world output. Since all products from foreign country l are initially purchased at export price, $P_{\mathrm{lt}}^{X}$, the economy-specific import good price can be expressed as:$P_{\mathrm{lkt}}^{M}=e_{\mathrm{lkt}}P_{\mathrm{lt}}^{X}$, where $e_{\mathrm{lkt}}$ is the bilateral exchange rate between domestic country *k* and foreign country *l*.

#### 3.3.2 Export sector

The exporting firms are competitive and export a good $X_{\mathrm{kt}}$ that is a combination of domestic output and import content. The corresponding export price is given by:

$$P_{\mathrm{kt}}^{X}=exp\left(\varepsilon_{kt}^{X}\right){\left[\left(1-\varepsilon_{kt}^{M}s_{k}^{M,Z}\right){\left(P_{\mathrm{kt}}^{O}\right)}^{1-\sigma_{k}^{z}}+\varepsilon_{kt}^{M}s_{k}^{M,Z}{\left(P_{\mathrm{kt}}^{M}\right)}^{1-\sigma_{k}^{z}}\right]}^{\frac{1}{1-\sigma_{k}^{z}}},$$

where $\varepsilon_{\mathrm{kt}}^{X}$ captures an export-specific price shock.

### 3.4 Monetary and fiscal policy

#### 3.4.1 EA Taylor rule

The ECB sets the policy rate $i_{\mathrm{EAt}}$ in response to the annualised EA-wide inflation gap, $\pi_{\mathrm{EAt}}^{c,vat,QA}$, and the annualised EA output gap:

$$i_{\mathrm{EA},t}-\bar{i}=\rho^{i}\left(i_{\mathrm{EA},t-1}-\bar{i}\right)+\left(1-\rho^{i}\right)\left[\eta_{\mathrm{EA}}^{i\pi }0.25\left(\pi_{\mathrm{EA},t}^{c,vat,QA}-{\bar{\pi }}_{\mathrm{EA}}^{c,vat,QA}\right)+\eta_{\mathrm{EA}}^{\mathrm{iy}}\left(log\left(0.25\sum_{r=1}^{4}Y_{\mathrm{EA}t-r}\right)-log\left(0.25\sum_{r=1}^{4}Y_{\mathrm{EA},t-r}^{\mathrm{pot}}\right)\right)\right]+\varepsilon_{\mathrm{EA},t}^{i},$$

where $\bar{i}=r+{\bar{\pi }}^{Y}$ is the steady-state nominal interest rate, equal to the sum of the steady-state real interest rate and GDP inflation in the steady state. The policy parameters ($\rho^{i}$, $\eta^{i\pi }$, $\eta^{\mathrm{iy}}$) capture the interest rate inertia and the response to the annualised inflation and output gaps, respectively. $\varepsilon_{\mathrm{EA},t}^{i}$ captures unexpected monetary policy changes.

#### 3.4.2 Member State fiscal policy

The government collects taxes on labour, $\tau_{k}^{N}$, capital, $\tau_{\;}^{K}$, and consumption, $\tau_{\;}^{C}$, as well as lump-sum taxes, ${\mathrm{tax}}_{\mathrm{kt}}^{\;}$, and constant excise duties on oil imports from RoW, $\tau_{\;}^{\mathrm{Oil}}P_{\;}^{Y0}$, and it issues one-period bonds, $B_{\mathrm{kt}}^{g}$. Government spending includes public consumption, $G_{\mathrm{kt}}^{\;}$, public investment, $I_{\mathrm{kt}}^{G}$, transfers, $T_{\mathrm{kt}}^{\;}$, and the servicing of the outstanding debt. $G_{\mathrm{kt}}^{\;}$, $I_{\mathrm{kt}}^{G}$, and $T_{\mathrm{kt}}^{\;}$ follow autoregressive processes with shocks $\varepsilon_{\mathrm{kt}}^{G}$:

$$\frac{G_{\mathrm{kt}}}{P_{\mathrm{kt}}^{Y}Y_{\mathrm{kt}}}-\bar{G}=\rho_{k}^{G}\left(\frac{G_{\mathrm{kt}-1}}{P_{\mathrm{kt}-1}^{Y}Y_{\mathrm{kt}-1}}-\bar{G}\right)+\varepsilon_{\mathrm{kt}}^{G},$$

where $G\in \left\{G,I^{G},T\right\}.$ The government budget constraint is:

$$B_{\mathrm{kt}}^{g}=\left(1+i_{\mathrm{kt}-1}^{g}\right)B_{\mathrm{kt}-1}^{g}-R_{\mathrm{kt}}^{G}+P_{\mathrm{kt}}^{G}G_{\mathrm{kt}}+P_{\mathrm{kt}}^{\mathrm{IG}}I_{\mathrm{kt}}^{G}+T_{\mathrm{kt}},$$

where nominal government revenues, $R_{\mathrm{kt}}^{G}$, are:

$$R_{\mathrm{kt}}^{G}=\tau_{\;}^{K}\left(P_{\mathrm{kt}}^{Y}Y_{\mathrm{kt}}-W_{\mathrm{kt}}N_{\mathrm{kt}}-P_{\mathrm{kt}}^{I}\delta_{k}K_{\mathrm{kt}-1}\right)+\tau_{k}^{N}W_{\mathrm{kt}}N_{\mathrm{kt}}+\tau_{\;}^{C}P_{\mathrm{kt}}^{C}C_{\mathrm{kt}}+\tau_{\;}^{\mathrm{Oil}}P_{\;}^{Y0}{\mathrm{Oil}}_{t}+tax_{kt}.$$

The government uses lump-sum taxes as a budget closure and increases (lowers) them when government debt and deficit are above (below) the respective targets, ${\bar{B}}_{k}^{g}$ and ${\mathrm{DEF}}_{k}^{T}$:

$$\frac{{\mathrm{tax}}_{\mathrm{kt}}}{{\bar{P_{\mathrm{kt}}^{Y}Y}}_{\mathrm{kt}}}=\rho^{\mathrm{tax}}\left(\frac{{\mathrm{tax}}_{\mathrm{kt}-1}}{{\bar{P_{\mathrm{kt}}^{Y}Y}}_{\mathrm{kt}}}\right)+\eta_{k}^{\mathrm{DEF}}\left(\frac{\Delta B_{\mathrm{kt}-1}^{g}}{P_{\mathrm{kt}-1}^{Y}Y_{\mathrm{kt}-1}}-{\mathrm{DEF}}_{k}^{T}\right)+\eta_{k}^{B}\left(\frac{B_{\mathrm{kt}-1}^{g}}{P_{\mathrm{kt}-1}^{Y}Y_{\mathrm{kt}-1}}-{\bar{B}}_{k}^{g}\right)+\varepsilon_{\mathrm{kt}}^{\mathrm{tax}}.$$

### 3.5 The trade balance and aggregate accounting

Market clearing requires that:

$$Y_{\mathrm{kt}}P_{\mathrm{kt}}^{Y}+\tau_{t}^{\mathrm{Oil}}{\mathrm{Oil}}_{\mathrm{kt}}P_{\;}^{Y0}=P_{\mathrm{kt}}^{C}C_{\mathrm{kt}}+P_{\mathrm{kt}}^{I}I_{\mathrm{kt}}+P_{\mathrm{kt}}^{G}G_{\mathrm{kt}}+P_{\mathrm{kt}}^{\mathrm{IG}}{\mathrm{IG}}_{\mathrm{kt}}+{\mathrm{TB}}_{\mathrm{kt}},$$

where the trade balance, ${\mathrm{TB}}_{\mathrm{kt}}$, is defined as the difference between nominal exports and imports, with domestic importers buying the imported good at the price $e_{lkt}P_{\mathrm{lt}}^{X}$:

$${\mathrm{TB}}_{\mathrm{kt}}\equiv P_{\mathrm{kt}}^{X}X_{\mathrm{kt}}-\sum_{l}\frac{{\mathrm{size}}_{l}}{{\mathrm{size}}_{k}}e_{\mathrm{lkt}}P_{\mathrm{lt}}^{X}M_{\mathrm{lkt}}-e_{\mathrm{RoW},EA,t}P_{\mathrm{RoW},t}^{\mathrm{Oil}}{\mathrm{OIL}}_{kt}$$

Exports are the sum of imports by other countries from the domestic economy, i.e. $X_{\mathrm{kt}}=\sum_{l}M_{\mathrm{lkt}}$, where $M_{\mathrm{lkt}}$ stands for imports of economy *l* from the domestic economy *k*. Total imports are:

$$P_{\mathrm{kt}}^{\mathrm{Mtot}}M_{\mathrm{kt}}^{\mathrm{tot}}=P_{\mathrm{kt}}^{M}M_{\mathrm{kt}}+e_{RoW,EA,t}P_{RoWt}^{\mathrm{oil}}{\mathrm{OIL}}_{\mathrm{kt}},$$

where non-oil imports are $P_{\mathrm{kt}}^{M}M_{\mathrm{kt}}=P_{\mathrm{kt}}^{M}\left(M_{\mathrm{kt}}^{C}+M_{\mathrm{kt}}^{I}+M_{\mathrm{kt}}^{G}+M_{\mathrm{kt}}^{\mathrm{IG}}\right)$. Net foreign assets (NFA), $B_{\mathrm{kt}}^{bw}$, evolve according to:^8^

$$e_{\mathrm{RoW},kt}B_{kt}^{bw}=\left(1+i_{t-1}^{\mathrm{bw}}\right)e_{\mathrm{RoW},kt}B_{kt-1}^{bw}+{\mathrm{TB}}_{\mathrm{kt}}+{\mathrm{ITR}}_{k}P_{\mathrm{kt}}^{Y}Y_{\mathrm{kt}},$$

where ITR_k_ are net international transfers received as share of GDP, introduced to align a non-zero trade balance with a stable NFA position in steady state. The NFA positions sum to zero at the global level, i.e. $NFA_{kt}size_{k}+NFA_{REA,t}size_{REA}+NFA_{RoW,t}size_{RoW}=0$.

### 3.6 The REA and RoW blocks

The REA and RoW (subscript *k* = REA, RoW) model blocks follow a simplified structure. They consist of a budget constraint for the representative household, demand functions for domestic and imported goods, a linear production technology, a New Keynesian Phillips curve, and a Taylor rule. The REA and RoW blocks abstract from capital accumulation.^9^ There are shocks to labour productivity, price mark-ups for final output, the subjective discount rate, the relative preference for domestic vs. imported goods, as well as monetary policy shocks.

The budget constraint for the representative household in REA, as an oil importer, is:

$$P_{\mathrm{REA},t}^{Y}Y_{\mathrm{REA},t}+\tau^{\mathrm{Oil}}P_{\;}^{Y0}{\mathrm{Oil}}_{\mathrm{REA},t}=P_{\mathrm{REA},t}^{C}C_{\mathrm{REA},t}+{\mathrm{TB}}_{\mathrm{REA},t},$$

where $\tau^{\mathrm{Oil}}P_{\;}^{Y0}{\mathrm{Oil}}_{\mathrm{REAt}}$ captures the excise duty.^10^ Total nominal exports of final goods for REA and RoW are defined as: $P_{\mathrm{kt}}^{X}X_{\mathrm{kt}}=\sum_{l}\frac{size_{l}}{size_{k}}P_{\mathrm{lkt}}^{X}M_{\mathrm{lkt}}$, with the bilateral export price being defined as the domestic price subject to a bilateral price shock, $P_{\mathrm{lkt}}^{X}=exp\left(\varepsilon_{\mathrm{lkt}}^{X}\right)P_{\mathrm{kt}}^{Y}$.

We combine the FOCs of REA and RoW with respect to international bonds to obtain the uncovered interest parity (UIP) condition:

$$E_{t}\left[\frac{e_{\mathrm{RoW},EA,t+1}}{e_{\mathrm{RoW},EA,t}}\right]\left(1+i_{\mathrm{RoW},t}\right)=\left(1+i_{\mathrm{EA},t}\right)+\varepsilon_{\mathrm{EA},t}^{\mathrm{bw}}+\alpha_{\mathrm{EA}}^{bw0}+\alpha_{\mathrm{EA}}^{bw1}\frac{e_{\mathrm{RoW},EA,t}B_{\mathrm{EA},t}^{bw}}{P_{\mathrm{EA},t}^{Y}Y_{\mathrm{EA},t}}$$

where $\varepsilon_{\mathrm{EA},t}^{\mathrm{bw}}$ captures a bond premium shock between EA and RoW (exchange rate shock), and $\alpha_{\mathrm{EA}}^{bw1}$ is a debt-dependent country risk premium on NFA holdings.^11^

In the absence of investment and government spending in the REA and RoW blocks, final domestic demand, $C_{\mathrm{kt}}$, is a CES aggregate of domestic output, $Y_{\mathrm{kt}}$, and imported goods, $M_{\mathrm{kt}}^{\;}$:

$$C_{\mathrm{kt}}=A_{\mathrm{kt}}^{p}{\left[{\left(1-\varepsilon_{kt}^{M}s_{k}^{M}\right)}^{\frac{1}{\sigma_{k}^{c}}}{\left(Y_{\mathrm{kt}}^{}\right)}^{\frac{\sigma_{k}^{c}-1}{\sigma_{k}^{c}}}+{\left(\varepsilon_{kt}^{M}s_{k}^{M}\right)}^{\frac{1}{\sigma_{k}^{c}}}{\left(M_{\mathrm{kt}}^{}\right)}^{\frac{\sigma_{k}^{c}-1}{\sigma_{k}^{c}}}\right]}^{\frac{\sigma_{k}^{c}}{\sigma_{k}^{c}-1}},$$

where $\varepsilon_{kt}^{M}s_{k}^{M}$ is the time-varying import share.

The intermediate good producers use labour to manufacture domestic goods :$Y_{\mathrm{kt}}=A_{\mathrm{kt}}^{Y}N_{\mathrm{kt}}$, where $A_{\mathrm{kt}}^{Y}$ captures a trend in productivity. Price setting follows a New Keynesian Phillips curve:

$$\pi_{\mathrm{kt}}^{Y}-{\bar{\pi }}_{k}^{Y}=\beta E_{t}\left[\frac{\lambda_{kt+1}^{\;}}{\lambda_{kt}^{\;}}\left[sfp_{k}\left(E_{t}\pi_{kt+1}^{Y}-{\bar{\pi }}_{k}^{Y}\right)+\left(1-sfp_{k}\right)\left(\pi_{kt-1}^{Y}-{\bar{\pi }}_{k}^{Y}\right)\right]\right]+\varphi_{k}^{Y}\ln\left(Y_{\mathrm{kt}}-{\bar{Y}}_{k}\right)+\varepsilon_{\mathrm{kt}}^{\mathrm{MUY}}$$

where $\lambda_{\mathrm{kt}}^{\;}=\Theta_{\mathrm{kt}}{\left(C_{\mathrm{kt}}-h_{k}C_{\mathrm{kt}-1}\right)}^{-\theta_{k}}$ is the marginal utility of consumption, ${\mathrm{sfp}}_{k}$ is the share of forward-looking price-setters, and $\varepsilon_{\mathrm{kt}}^{\mathrm{MUY}}$ is a cost-push shock.

The intertemporal equation for aggregate domestic demand follows from the FOC for consumption:

$$\beta E_{t}\left[\frac{\lambda_{kt+1}^{\;}}{\lambda_{kt}^{\;}}\frac{1+i_{kt}}{1+\pi_{kt+1}^{C}}\right]=1$$

with $\frac{\lambda_{kt+1}}{\lambda_{kt}}$ featuring $\varepsilon_{\mathrm{kt}}^{c}$ as the REA and RoW demand shock, respectively.

Monetary policy in RoW follows a Taylor-type rule similar to the EA (estimated parameters are region-specific).

## 4 Model solution and econometric approach

The following non-linear system summarizes the state-space representation of our model:

$$E_{t}\left[F\left(y_{t+1},y_{t},y_{t-1},\varepsilon_{t};\theta \right)\right]=0,$$

where $y_{t}$ collects all endogenous variables of the model, while $\varepsilon_{t}$ is a vector of exogenous shocks. We compute an approximate model solution by linearising the model around its deterministic steady state. Given the structural parameters collected in θ, the linear rational expectation solution takes the following form:

$$y_{t}=\Phi_{1}\left(\theta \right)+\Phi_{\varepsilon }\left(\theta \right)\varepsilon_{t},$$

where $\Phi_{1}$ and $\Phi_{\varepsilon }$ govern the decision rules of the model.

We calibrate a subset of parameters to match long-run data properties, and we estimate the remaining parameters with Bayesian methods using data for the period 1999q1-2018q4. To perform a large number of robustness checks, we use a computationally efficient parallelised slice sampling algorithm.^12^ Appendix C provides information on data transformations and our data set.

The calibration of parameters for the long run replicates average historical ratios and trade shares for the respective MS (see Table B1 in the appendix). All real GDP components on the demand side (deflated by the GDP deflator) are assumed to grow at the average growth rate of output over the sample period. Prices in steady state grow at a rate of 2% per year. We set the steady-state share of Ricardian households according to the survey evidence in Dolls et al. (2012). The parameters of the EA monetary policy rule have been estimated in a two-region (EA-RoW) version of the model and are imposed here to ensure an identical policy rule for both EA configurations, i.e. DE-REA-RoW and ES-REA-RoW.

Table 1 presents the chosen priors and posterior estimates for key parameters. Consumption habit persistence of around 0.7 in DE and ES suggests relatively sluggish adjustment of consumption demand to changes in income. The model estimation indicates a slightly higher risk aversion and labour supply elasticity in ES. Aggregate import price elasticities are estimated at 1.5 and 1.2 in DE and ES, respectively. Lower employment adjustment costs in ES relate to the highly cyclical unemployment dynamics observed in the last two decades. The estimates also suggest substantial nominal rigidities in prices and wages.

**Table 1:** Selected estimated model parameters.

		Prior distribution		Posterior distribution	
		Distr	Mean		
			St.dev.	DE	ES
***Preferences***					
Consumption habit persistence	h	Beta	0.5	0.68	0.70
			0.1	(0.67, 0.80)	(0.68, 0.81)
Risk aversion	θ	Gamma	1.5	1.45	1.75
			0.2	(1.18, 1.80)	(1.39, 1.96)
Share of forward-looking wage setters	sfw	Normal	1	0.75	0.77
			0.5	(0.47, 0.90)	(0.32, 0.89)
Inverse Frisch elasticity	$\theta^{N}$	Gamma	2.5	2.43	2.23
			0.5	(1.83, 3.14)	(1.45, 2.58)
Share of forward-looking price setters	sfp	Normal	1	0.98	0.98
			0.5	(0.89, 1.00)	(0.93, 1.00)
Elasticity of substitution of imports	$\sigma^{z}$	Gamma	2	1.48	1.21
			0.4	(1.13, 1.49)	(1.11, 1.40)
Bilateral price elasticity of imports	$\sigma^{\mathrm{FM}}$	Gamma	2	2.00	0.85
			1	(0.57,2.86)	(0.37,2.44)
Oil price elasticity	$\sigma^{O}$	Gamma	0.5	0.19	0.25
			0.2	(0.02, 0.33)	(0.03, 0.42)
***Nominal and real frictions***					
Price adjustment cost	$\gamma^{P}$	Gamma	60	28.33	22.42
			40	(12.62, 38.30)	(14.70, 29.70)
Nominal wage adjustment cost	$\gamma^{w}$	Gamma	5	4.40	1.53
			2	(2.64, 6.44)	(1.41, 3.77)
Real wage rigidity	$\gamma^{\mathrm{wr}}$	Beta	0.5	0.96	0.97
			0.2	(0.95, 0.98)	(0.97, 0.99)
Employment adjustment cost	$\gamma^{n}$	Gamma	60	41.68	10.07
			40	(15.78, 66.53)	(5.17, 19.90)
Labour hoarding adjustment cost	$\gamma^{\mathrm{fn},2}$	Gamma	2	1.52	1.30
			0.5	(1.21, 1.84)	(1.08, 1.52)
Capacity utilisation adj. cost	$\gamma^{u,2}$	Gamma	0.003	0.004	0.004
			0.0012	(0.002, 0.006)	(0.001, 0.004)
Capital stock adjustment cost	$\gamma^{I,1}$	Gamma	60	46.68	34.49
			40	(33.39, 65.58)	(17.92, 51.88)
Investment adjustment cost	$\gamma^{I,2}$	Gamma	60	9.66	55.93
			40	(0.98, 19.90)	(51.96, 128.75)
***Fiscal policy***					
Lump-sum tax persistence	$\rho^{\mathrm{tax}}$	Beta	0.5	0.88	0.94
			0.2	(0.90, 0.97)	(0.92, 0.98)
Lump-sum tax response to deficit	$\eta_{\;}^{\mathrm{DEF}}$	Beta	0.03	0.02	0.03
			0.008	(0.01, 0.04)	(0.02, 0.04)
Lump-sum tax response to debt	$\eta_{\;}^{B}$	Beta	0.02	0.003	0.003
			0.01	(0.001, 0.006)	(0.002, 0.005)
***REA region***					
Consumption habit persistence	h	Beta	0.7	0.87	0.84
			0.1	(0.80, 0.89)	(0.79, 0.89)
Risk aversion	θ	Gamma	1.5	1.49	1.37
			0.2	(1.22, 1.78)	(1.25, 1.79)
Import price elasticity	$\sigma^{C}$	Gamma	2	1.37	1.39
			0.4	(1.10, 1.40)	(1.11, 1.35)
Phillips curve slope	$\varphi^{Y}$	Gamma	0.025	0.03	0.03
			0.01	(0.02, 0.05)	(0.02, 0.05)
Share of forward-looking price setters	sfp_REA_	Normal	1	0.79	0.72
			0.5	(0.38, 0.82)	(0.17, 0.72)
***RoW region***					
Consumption habit persistence	h	Beta	0.7	0.92	0.88
			0.1	(0.88, 0.93)	(0.87, 0.92)
Risk aversion	θ	Gamma	1.5	1.31	1.64
			0.2	(1.28, 2.05)	(1.37, 2.04)
Import price elasticity	$\sigma^{C}$	Gamma	2	1.38	1.39
			0.4	(1.14, 1.54)	(1.16, 1.59)
Phillips curve slope	$\varphi^{Y}$	Gamma	0.025	0.01	0.05
			0.01	(0.01, 0.05)	(0.01, 0.06)
Share of forward-looking price setters	*sfp*_RoW_	Normal	1	0.25	0.90
			0.5	(0.01, 0.60)	(0.03, 0.92)

Note: Cols. (1)-(2) list model parameters. Cols. (3)-(4) indicate the prior distribution function. Identical priors are assumed for DE and ES parameters. Cols. (5)-(8) show the mode and the (10% and 90%) HPD intervals of the posterior distributions.

Demand shocks are highly serially correlated, as shown in Table 2.^13^ Table B3 and Table B4 in Appendix B.2 shows that model-implied moments are close to the data and that the estimated model successfully replicates business cycles features in DE and ES.

**Table 2:** Selected estimated exogenous shock processes.

		Prior distribution		Posterior distribution	
		Distr	Mean		
			St.dev.	DE	ES
***Autocorrelation of forcing variables***					
Subjective discount factor	$\rho^{c}$	Beta	0.5	0.87	0.84
			0.2	(0.81, 0.91)	(0.81, 0.89)
Investment risk premium	$\rho^{S}$	Beta	0.85	0.90	0.94
			0.05	(0.87, 0.95)	(0.94, 0.97)
Domestic price mark-up	$\rho^{\mathrm{MUY}}$	Beta	0.5	0.67	0.21
			0.2	(0.54, 0.76)	(0.14, 0.59)
Labour supply	$\rho^{U}$	Beta	0.5	0.90	0.88
			0.2	(0.87, 0.96)	(0.80, 0.92)
Flight-to-safety	$\rho^{\mathrm{FQ}}$	Beta	0.85	0.96	0.96
			0.05	(0.94, 0.99)	(0.92, 0.98)
Trade share	$\rho^{M}$	Beta	0.5	0.87	0.89
			0.2	(0.86, 0.96)	(0.84, 0.94)
Export price	$\rho^{X}$	Beta	0.5	0.97	0.86
			0.2	(0.96, 0.99)	(0.86, 0.96)
International bond preferences	$\rho^{\mathrm{BW}}$	Beta	0.5	0.94	0.90
			0.2	(0.88, 0.94)	(0.85, 0.95)
Government consumption	$\rho^{G}$	Beta	0.5	0.96	0.92
			0.2	(0.91, 0.96)	(0.90, 0.93)
Government transfers	$\rho^{T}$	Beta	0.5	0.96	0.96
			0.2	(0.95, 0.98)	(0.94, 0.97)
Government investment	$\rho^{\mathrm{IG}}$	Beta	0.7	0.85	0.94
			0.1	(0.78, 0.90)	(0.92, 0.97)
Government tax	$\rho^{\mathrm{TAX}}$	Beta	0.5	0.88	0.94
			0.2	(0.88, 0.97)	(0.92, 0.98)
Permanent TFP growth	$\rho^{\mathrm{GAY}}$	Beta	0.5	0.96	0.97
			0.2	(0.94, 0.97)	(0.95, 0.98)
***Standard deviation (%) of innovations to forcing variables***					
Subjective discount factor	$\varepsilon^{c}$	Gamma	1	0.74	1.00
			0.4	(0.51, 1.34)	(0.63, 1.37)
Investment risk premium	$\varepsilon^{S}$	Gamma	0.1	0.20	0.23
			0.04	(0.14, 0.31)	(0.17, 0.31)
Price mark-up	$\varepsilon^{\mathrm{MUY}}$	Gamma	2	5.70	5.80
			0.8	(3.10, 6.40)	(4.10, 7.10)
Labour supply	$\varepsilon^{U}$	Gamma	1	1.40	1.50
			0.4	(0.86, 1.97)	(1.50, 3.16)
Flight-to-safety	$\varepsilon^{\mathrm{FQ}}$	Gamma	1	0.08	0.07
			0.04	(0.07, 0.10)	(0.06, 0.09)
Trade share	$\varepsilon^{M}$	Gamma	1	2.50	2.40
			0.4	(2.00, 2.52)	(2.20, 2.91)
Export price	$\varepsilon^{X}$	Gamma	1	0.34	0.64
			0.4	(0.31, 0.42)	(0.61, 0.80)
International bond preferences	$\varepsilon^{\mathrm{BW}}$	Gamma	1	0.14	0.23
			0.4	(0.15, 0.28)	(0.14, 0.34)
Government consumption	$\varepsilon^{G}$	Gamma	1	0.12	0.11
			0.4	(0.11, 0.16)	(0.11, 0.13)
Government transfers	$\varepsilon^{T}$	Gamma	1	0.13	0.23
			0.4	(0.11, 0.14)	(0.19, 0.25)
Government investment	$\varepsilon^{\mathrm{IG}}$	Gamma	1	0.07	0.24
			0.4	(0.06, 0.08)	(0.23, 0.29)
Government tax	$\varepsilon^{\mathrm{TAX}}$	Gamma	1	0.62	1.04
			0.4	(0.53, 0.65)	(0.97, 1.27)
Permanent TFP growth	$\varepsilon^{\mathrm{GAY}}$	Gamma	0.01	0.006	0.010
			0.004	(0.005, 0.007)	(0.001, 0.003)
Permanent TFP level	$\varepsilon^{\mathrm{LAY}}$	Gamma	0.01	0.002	0.002
			0.004	(0.001, 0.002)	(0.001, 0.003)
EA monetary policy	$\varepsilon^{i}$	Gamma	1	0.097	0.090
			0.4	(0.080, 0.099)	(0.082, 0.109)

Note: Cols. (1)-(2) list model innovations. Cols. (3)-(4) indicate the prior distribution function. Identical priors are assumed for DE and ES parameters. Cols. (5)-(8) show the mode and the (10% and 90%) HPD intervals of the posterior distributions.

## 5 Estimated drivers of the trade balance in the benchmark model

**Fig. 3:**
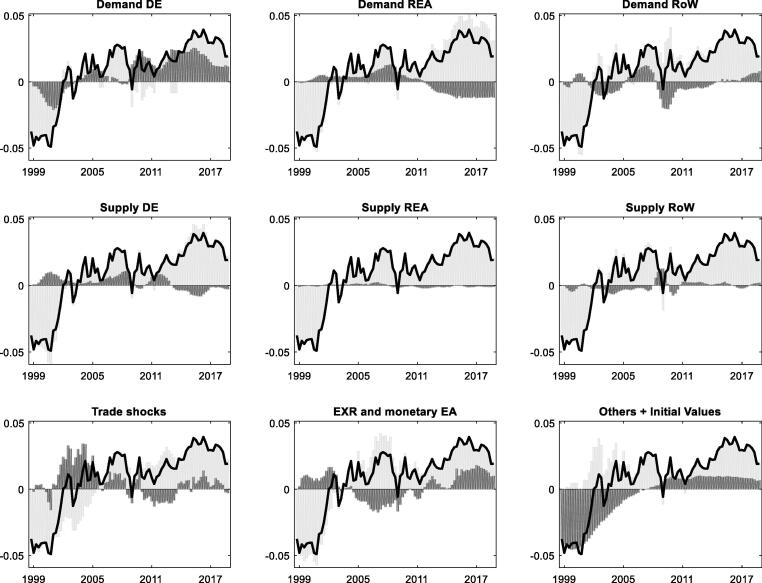
**Shock decomposition of the German trade balance-to-GDP ratio** Note: Units on the x-axis are years and units on the y-axis measure the trade balance as a share of GDP relative to its sample mean, where 0.01 corresponds to 1% of GDP. The mean (steady state) of the trade balance-to-GDP ratio in DE is 4.0%. The solid lines represent the historical series of the trade balance-to-GDP ratio from which we have subtracted the sample average. Vertical bars measure the estimated contribution of different shock groups. Bars above (below) the x-axis indicate positive (negative) contributions to the trade balance relative to its average in a given year. The sum of positive and negative contributions matches the data (solid black line) for any point in time. We have assigned shocks to distinct groups, mainly focusing on demand versus supply shocks originating in different regions (domestic, REA, and RoW). In addition, we report shocks to preferences for foreign goods and mark-up shocks to import and export prices as “trade shocks”, and shocks to EA monetary policy and the interest rate parity condition between the EA and the RoW as “EXR and monetary policy shocks.” The group “Others + Initial Values” summarizes any remaining factors and the effect of initial conditions. Initial conditions (“initial disbalances”) are estimated measures of how much the starting values in the data deviate from the model steady state.

**Fig. 4:**
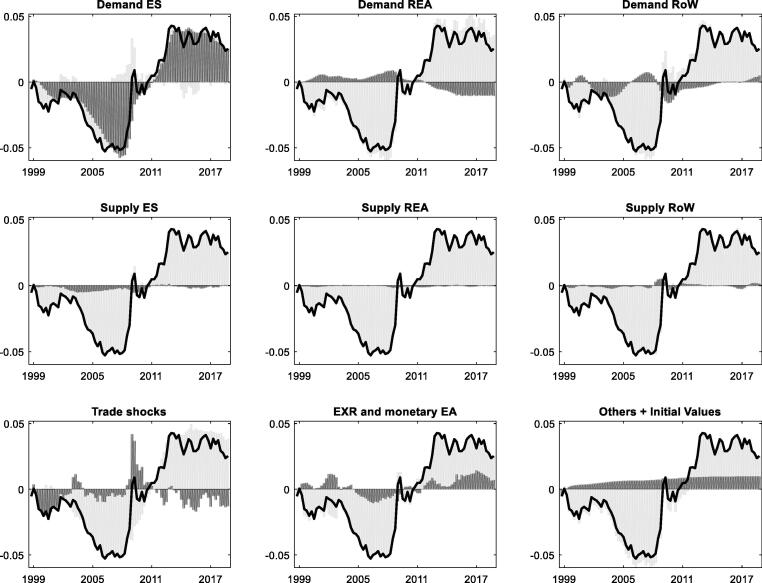
**Shock decomposition of the Spanish trade balance-to-GDP ratio** Note: Units on the x-axis are years and units on the y-axis measure the trade balance as a share of GDP relative to its sample mean, where 0.01 corresponds to 1% of GDP. The mean (steady state) of the trade balance-to-GDP ratio in ES is −1.1%. For additional information see also the description below Fig. 3.

This section quantifies the main drivers of the TBs of DE and ES based on the estimated benchmark model. Fig. 3 and Fig. 4 assess the role of different shocks as drivers of the TB for DE and ES, respectively. We first consider the historical decomposition of DE TB as the EA's emblematic surplus country in Fig. 3. The estimates suggest that domestic demand conditions, namely excess saving and adverse investment shocks, account for a large share of the surplus build-up. The shocks are very persistent, which explains the non-cyclical upward trend in Fig. 1 above. Exchange rate shocks (euro depreciation) in recent years have also contributed to the surplus, as have trade shocks, notably higher demand for DE goods and services (preference shift). Positive trade shocks cannot explain the persistent upward trend in DE TB, however, given that the former have also been present (and even stronger) in the early 2000s, before switching sign in the Global financial crisis and recession that saw a pronounced slowdown in world trade. Strong (weak) aggregate demand in the REA has strengthened (lowered) DE TB before (during and after) the EA crisis.

The global recession as a negative shock to RoW demand has been an important driver behind the decline in DE TB in 2009, and the RoW recovery has contributed to its renewed rise in the 2010s. The positive contribution by domestic supply shocks reflects competitiveness gains from the labour market (“Hartz”) reforms after 2004–05 and wage moderation, but the effect is temporary.^14^ External supply factors (REA and RoW) have little impact on DE TB and do not contribute to explaining the main pattern of a rising TB over the sample horizon.

The dynamics of the ES TB is more cyclical, as illustrated in Fig. 1 above. The TB has displayed a large and growing deficit during the first decade of EMU and sharp imbalance correction afterwards. The historical decomposition in Fig. 4 shows that the ES TB has been primarily driven by the boom-bust cycle of domestic demand, according to the model estimates. The loosening of credit constraints, asset price bubbles, and the construction boom led to strong import demand and pronounced TB deficits in the pre-crisis period. Subsequently, the contraction of domestic demand and the double-dip recession have led to a sharp TB reversal. The results suggest that the ES TB (in % of nominal GDP) would have been more than 5 pp higher in 2008 in the absence of positive domestic demand shocks, and the subsequent reversal about 4 pp weaker. Foreign (REA and RoW) demand has played a similar role as in the case of DE, and foreign supply shocks likewise have little impact. Trade shocks are noticeable, but, as for DE, they do not explain the overall pattern of the ES TB. The role of euro exchange rate has been similar to DE's case and has supported the ES TB reversal after the global recession and EA crisis.

Appendix B.3 provides further details on the role of different shocks in the “domestic demand” group and stresses the minor role of the estimated fiscal shocks in the TB decompositions. In line with Giovannini et al. (2019), it also shows that oil price shocks weigh negatively on the DE and ES TBs, when the shocks correspond to a price increase, and positively, when the shocks imply a decline in oil prices, as, e.g., in (2015–17), and an associated decline in the import bill. The contribution of oil price shocks to DE and ES TB dynamics remains modest, however, compared to the impact of, notably, domestic demand shocks.

In sum, the estimated benchmark version of the model suggests that spillover from foreign shocks, particularly foreign demand shocks, has played a notable but limited role for TB dynamics in DE and ES. The estimation provides little evidence for quantitatively important supply-driven spillover, such as exogenous “competitive gains/losses” or “competitive pressure”, on the TBs of DE and ES. The rest of the paper examines the secondary role of foreign shocks in more detail in a more agnostic setting.

## 6 Inspecting the robustness of the benchmark results

The estimated benchmark specification suggests that domestic demand conditions are the key drivers of DE and ES TBs, whereas foreign shocks, and in particularly foreign supply-side shocks, play a much smaller role. The estimated model thus supports the hypothesis of primarily demand- and domestically-driven TB dynamics in the two countries. This section presents econometric tests to assess the robustness of this result. Section 6.1 directly includes domestic and foreign mark-up shocks in the trade equations to allow for a stronger presence of competitiveness effects in trade, reflecting the idea that price and wage pressure from abroad may have affected net exports of EA Member States more strongly than foreseen by the standard trade equations, so that competitive pressure may explain part of the benchmark model’s trade shocks. Section 6.2 enriches the empirical specification more generally. It augments the model with agnostic structural disturbances (ASDs), proposed by Den Haan and Drechsel (2021), as a “theory-free” alternative to structural shocks (“drivers”) that we identified as drivers in Section 5.

### 6.1 Strenghtening the competitiveness channel

This subsection inspects the role of price and wage dynamics for the TB. We extend the benchmark specification by including shocks to price and wage dynamics directly in the behavioural equations describing import and export dynamics. Price and wage setting equations remain identical to the benchmark model. We then re-estimate the model to test for a direct impact of price and wage shocks in the trade equations that goes beyond the impact via current prices. We consider four different specifications for each country, i.e. a total of eight estimated model versions. The first specification looks at domestic wage mark-up shocks and their potential to explain trade, notably exports. The second set-up does the same for domestic price mark-up shock. For completeness, we allow the domestic shocks to also enter REA and RoW trade equations directly in the first two variants. The two remaining experiments incorporate, respectively, REA and RoW price mark-up shocks in the trade equations to allow for more direct effects of competitive pressure. Specifically, DE and ES export and import equations include the REA and RoW mark-up shocks ($\varepsilon_{\mathrm{kt}}^{\mathrm{MUY}}$ with k∈{REA,RoW}) as additional additive factors in these experiments. The procedure is agnostic in the sense of allowing structural disturbances to enter additional model equations without constraining the sign and size of additional effects and, hence, letting the data speak more freely. We assign wide priors with zero mean such that the four variants remain *a priori* identical to the benchmark specification of Section 5.

Table 3 provides the data density of the four variants as the criterion for model selection in the Bayesian context. The data density evaluates the fit of the model, but also penalizes models with more parameters, giving a preference to simplicity.^15^ Table 3 shows that the data reject all four augmented specifications for the case of ES, i.e. supports the benchmark results that spillover from supply-side shocks have played little role for ES TB dynamics. Interestingly, however, the estimation favours a reinforced role for foreign (REA and RoW) and domestic price pressure in the case of DE, whereas the data reject the inclusion of the domestic wage mark-up also for the DE model.

**Table 3:** Data density of models with augmented trade equations.

	DE	ES
Benchmark model	11571.80	10804.74
REA price markup	11573.79	10802.54
RoW price markup	11573.91	10804.33
Domestic price markup	11590.25	10798.94
Domestic wage markup	11563.51	10795.16

Note: The data density is reported in log points using a Laplace approximation.

**Fig. 5:**
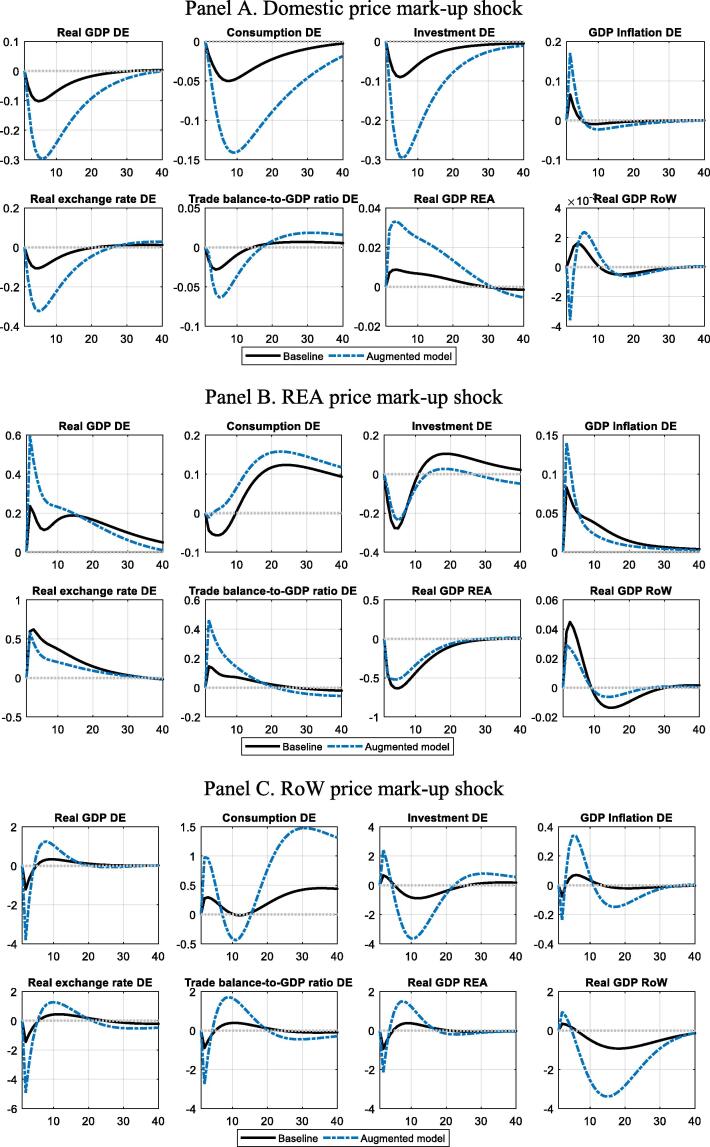
**Dynamics effects of augmented shocks in Germany** Panel A. Domestic price markup shock Panel B. REA price markup shock Panel C. RoW price markup shock Note: Dynamic effects of markup shocks (shock sizes are normalised to 1% to ensure comparability of the transmission mechanism). Solid black lines refer to the benchmark model, dashed blue to the augmented model with price markup shocks in the trade equations. An increase in the real exchange rate corresponds to a real effective depreciation. Units on the x-axis are quarters, and units on the y-axis are percentage-point deviations from the steady state for the real interest rate, inflation, and the trade balance, and per-cent deviations from steady state for all other variables. (For interpretation of the references to colour in this figure legend, the reader is referred to the web version of this article.)

Fig. 5 shows impulse response functions (IRFs) for negative domestic and foreign price markup shocks (increase in prices relative to production costs) in the augmented versions of the DE model. The responses correspond to the benchmark model in qualitative terms, i.e. the transmission of the shocks remains essentially intact. The reactions of domestic and foreign economic activity are stronger in the augmented model, however, implying stronger spillover of competitiveness differentials to economic activity. Consistently with the reinforced transmission, REA and RoW supply shocks explain slightly more of the DE TB dynamics relative to the benchmark specification. Nonetheless, the overall share of foreign supply shocks in the DE TB decomposition remains small, which provides additional evidence in favour of the results from the estimated benchmark specifications for DE and ES.

### 6.2 Agnostic structural disturbances

This subsection generalises the empirical specification further to soften model-imposed restrictions on the shock transmission. Based on the “agnostic structural disturbances” (ASD) approach of Den Haan and Drechsel (2021), it investigates whether estimated shocks that mainly drive the TB in the benchmark decompositions are correctly specified, or whether an alternative shock structure would change the relative importance of domestic and foreign shocks.

Formally, ASDs are structural shocks (disturbances) that enter the model like regular structural shocks. Their role in explaining the observed data is *a priori* unrestricted, however. We can rewrite the model representation as:

$$E_{t}\left[F\left(y_{t+1},y_{t},y_{t-1},\varepsilon_{t}^{R};\theta \right)\right]+\hat{Y}\varepsilon_{t}^{\mathrm{ASD}}=0,$$

where we partition the set of disturbances into regular shocks$,\varepsilon_{t}^{R}$, and the ASDs, $\varepsilon_{t}^{\mathrm{ASD}}.$ $\hat{Y}$ is an estimated vector of coefficients that determines the impact of the ASD on *all* model equations, with one coefficient for each model equation. A zero coefficient in a specific equation means that the ASD will have no impact on this equation. A coefficient different from zero implies that the ASD does enter the particular equation. Hence, ASDs enter the model without theoretical restrictions and may capture any “missing” shock. The tests conducted in Subsection 6.1 may be viewed as a “constrained” ASD procedure, where $\hat{Y}$ contains a few estimated non-zero entries, whereas the other coefficients are set to zero.^16^ In this section, by contrast, the ASDs enter the main behavioural equations. Once the coefficients in $\hat{Y}$ are estimated, we can assess the relevance of the respective ASD for macroeconomic and TB dynamics and interpret the ASD transmission from the point of economic theory to classify its nature.^17^

The ASD procedure is especially insightful when we replace a key shock from our baseline set-up. The replacing ASD enters the model in the same equation as the original shock *and* many other equations. The impact in *all* equations is *a priori* zero, including in the original location. In this way, the model estimation can detect potential misspecification of structural shocks. The estimation then agnostically determines the properties of the ASD. If, for instance, the resulting ASD assigns a large coefficient to the place of the original shock and the ASD behaves similarly to the omitted shock (similar IRFs), the procedure supports the original specification. Otherwise, the data may point to a “theory-free”, but econometrically preferred alternative shock structure. As in Subsection 6.1, we use the estimated data density to evaluate the fit of the model.

We focus the analysis on shocks to domestic demand and exogenous changes in competitiveness, which reflect competing hypotheses about the sources of external imbalances.^18^ For each shock replaced by an ASD, we re-estimate the model parameters and shock processes.

Table 4 shows that the data indeed prefer some of the agnostic specifications to the benchmark, suggesting that the extended models provide a better fit.

**Table 4:** Data density of augmented models (ASDs).

	DE	ES
Benchmark model	11571.80	10804.74
Flight-to-safety ASD	11585.42	10820.53
Wage markup ASD	11590.73	10808.48
Savings ASD	11563.54	10833.99
Investment risk premium ASD	11579.04	10814.97

Note: The data density is reported in log points using a Laplace approximation.

#### 6.2.1 Replacing a key domestic demand shock

The flight-to-safety shock is critical in the estimated benchmark model in Section 5, where it is the main driver of domestic demand and the TB-to-GDP ratio in ES (Fig. 4 above). Replacing this flight-to-safety shock by an ASD therefore opens the possibility of decomposing the TB dynamics in an entirely different way. The estimated ASD specification, however, shows the benchmark result to be robust to the modification. In particular, the decomposition of the TB-to-GDP ratio remains similar, supporting the benchmark estimates.

**Fig. 6:**
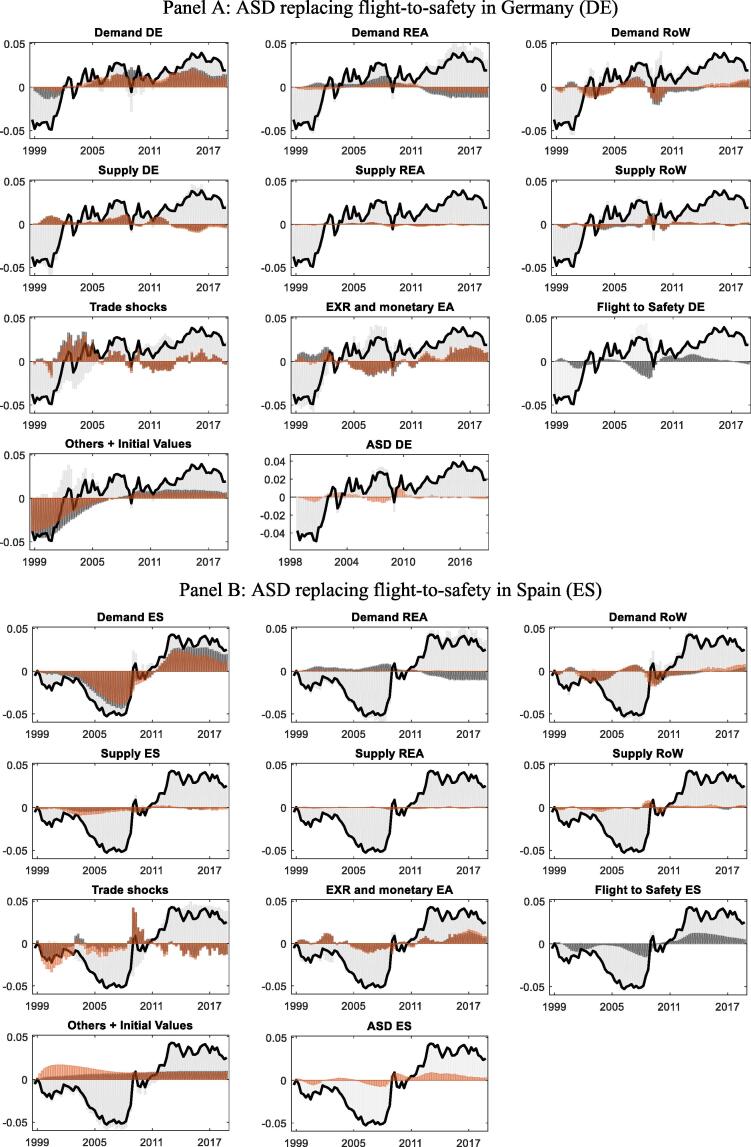
Trade balance-to-GDP ratio with “flight-to-safety” ASD Panel A: ASD replacing flight-to-safety in Germany (DE) Panel B: ASD replacing flight-to-safety in Spain (ES) Note: Units on the x-axis are years and units on the y-axis measure the trade balance (as share of GDP) relative to its sample mean, where 0.01 corresponds to 1% of GDP. Black bars show the contributions in the baseline version and red bars the contributions in the ASD specification. (For interpretation of the references to colour in this figure legend, the reader is referred to the web version of this article.)

Fig. 6 compares shock contributions of the benchmark model and the alternative ASD specification. The group of domestic demand shocks in the figure excludes the flight-to-safety shock in the benchmark model and the respective ASD in the ASD specification. While there are small quantitative differences, the relative importance of the different groups remains almost unchanged for both DE and ES. In particular, the original flight-to-safety shock and the replacing ASD shock display remarkably similar contributions for both countries, and the role of other shocks remain (largely) unchanged, thereby preserving the conclusions from the historical decomposition of the fully micro-founded model.

**Fig. 7:**
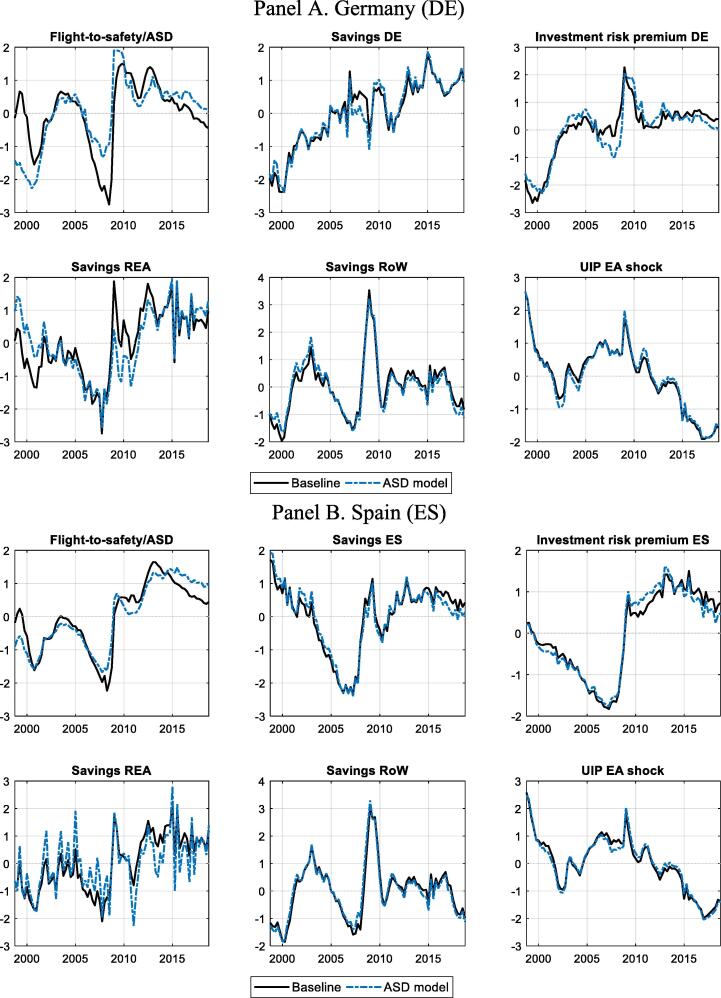
**Smoothed estimates of key demand shocks** Panel A. Germany (DE) Panel B. Spain (ES) Note: Solid black lines refer to the benchmark model and dashed blue ones to the ASD set-up. All shocks are standardised (to zero mean and standard deviation of one). (For interpretation of the references to colour in this figure legend, the reader is referred to the web version of this article.)

Fig. 7 shows that the main estimated demand shocks in the benchmark model and the ASD extension follow very similar paths. In particular, the estimated ASD closely follows the path of the flight-to-safety shock that it replaces, with higher persistence in recent years.

**Fig. 8:**
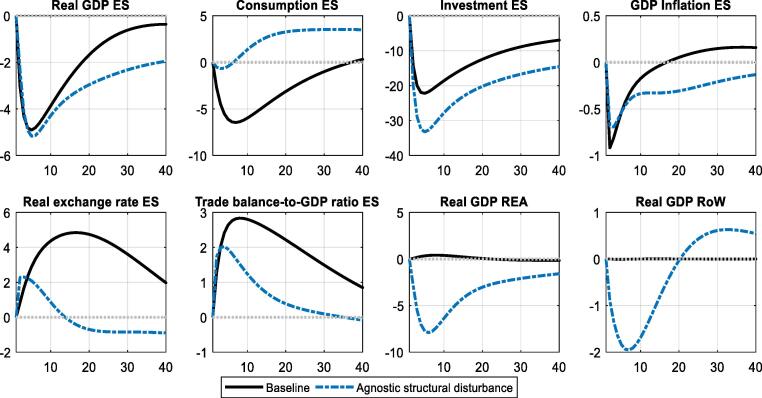
**IRFs for flight-to-safety and corresponding ASD in Spain (ES)** Note: Dynamic effects of flight-to-safety shocks (we normalize the shock size to 1%). Solid black lines refer to the benchmark model, dashed blue to the agnostic model. An increase in the real exchange rate corresponds to a real effective depreciation. Units on the x-axis are quarters, units on the y-axis are percentage-point deviations from the steady state (trade balance) and per cent (%) deviations from steady state (all other variables), respectively. (For interpretation of the references to colour in this figure legend, the reader is referred to the web version of this article.)

Does the transmission of the ASD resemble a domestic demand shock? Fig. 8 illustrates that the responses to this shock display strong similarity to the original flight-to-safety specification. At the same time, the ASD generates significant positive co-movement between domestic and foreign economic activity, in particular activity in REA. This co-movement indicates the estimated ASD shock to be more global than the original domestic flight-to-safety shock. It also leans more towards lower investment, to the benefit of higher consumption demand.

The literature on business cycle synchronisation has highlighted the role of common exposure to global risk factors (e.g., (Bacchetta and van Wincoop, 2013) and cross-country transmission of shocks to explain the international co-movement in economic activity, in particular with respect to the global financial crisis. Several papers have explained international synchronisation by global financial intermediation. Perri and Quadrini (2018), e.g., offer a theory based on financial frictions and self-fulfilling expectations.^19^ (Kalemli-Ozcan et al., 2013), Kollmann et. al. (2011), and Kollmann (2013) build on a microfounded propagation mechanism based on globally operating banks. Born and Enders (2019) support the role of financial frictions, but their calibration exercise suggests a dominance of the collapse in trade over financial factors in explaining DE output dynamics. Darracq, Paries and Papadopoulou (2019) calibrate a model with a rich set of financial frictions (banking, portfolio adjustment frictions, and bonds of different maturity) similar to Kollmann et al. (2015) to analyse (spillover effects of) non-conventional monetary policy. Sovereign default risk may further hamper financial intermediation. Bocola (2016), e.g., finds a sizable pass-through of sovereign risk to the private sector. The ASD estimates in this section support the idea of domestic demand conditions being linked to international (financial) factors.

#### 6.2.2 Replacing a key competitiveness shock

Wage moderation (wage markup) shocks have contributed to the DE TB surplus, notably during the period of the “Hartz” labour market reforms, according to the benchmark model. This section presents a model variant that replaces the wage markup shock by an ASD.

Table 4 suggests related improvements in the model fit for ES and, in particular, for DE.

**Fig. 9:**
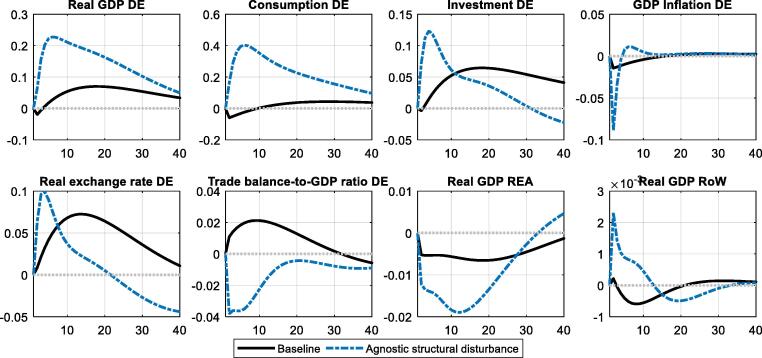
**IRFs for wage markup and replacing ASD shock in Germany** Note: Dynamic effects of wage markup shocks (we normalise the shock size to 1%). Solid black lines refer to the benchmark model, dashed blue ones to the agnostic model. An increase in the real exchange rate corresponds to a real effective depreciation. Units on the x-axis are quarters, units on the y-axis are percentage-point deviations from the steady state (trade balance) and per cent (%) deviations from steady state (all other variables), respectively. (For interpretation of the references to colour in this figure legend, the reader is referred to the web version of this article.)

The estimated IRFs in Fig. 9 characterise the ASD in the model for DE. The ASD replacing the wage markup shock combines characteristics of demand and supply shocks. Output increases in conjunction with a decline in the price level and REER depreciation, which is characteristic for positive supply shocks. At the same time, domestic demand increases sharply, driven notably by strong consumption demand from Ricardian households. TB falls in response to the ASD, contrary to the TB improvement in response to a negative wage markup shock in the benchmark model.

## 7 Conclusion

This paper examines the trade balance (TB) dynamics of Germany (DE) and Spain (ES), emblematic cases with very distinct TB dynamics since the start of EMU in 1999, in estimated multi-region open-economy DSGE models that feature rich trade linkages and international financial markets. In line with previous results from this class of models, the estimated benchmark models ascribes a large part of TB dynamics to domestic drivers, notably domestic demand shocks, although international (foreign demand) and supply factors also matter for the TB profile, particularly for DE. We revisit the benchmark result by adopting more agnostic approaches with respect to shock transmission and use the “agnostic structural disturbances” of Den Haan and Drechsel (2021). Letting the data speak more freely suggests that the benchmark model neglects elements of international co-movement, but these additional factors do not fundamentally alter the decomposition of the TB dynamics. The domestic (demand) drivers remain dominant also when theoretical restrictions on the shock transmission are relaxed.

The distinction between domestic versus foreign and demand versus supply shocks is, admittedly, less sharp in reality. Domestic demand shocks in the model reflect, e.g., financial constraints, such as credit supply by foreign and domestic financial intermediaries, and may be subject to financial contagion that is unrelated to the structural linkages included in the model. In this sense, positive (negative) private domestic demand shocks, notably in the model for ES, may, e.g., also relate to softening (tightening) financial constraints and strengthening (weakening) credit supply by foreign lenders.

## Declaration of Competing Interest

The authors declare that they have no known competing financial interests or personal relationships that could have appeared to influence the work reported in this paper.

## Figures and Tables

**Table B1:** Selected calibrated parameters.

**Parameters**		**DE**	**ES**
***Preferences***			
Intertemporal discount factor	β	0.998	0.998
Share of Ricardian households	$\omega^{s}$	0.610	0.690
Weight of disutility	$\omega^{N}$	1.095	4.340
Degree of openness	$s^{M}$	0.394	0.308
Preference for domestic risky assets	$\alpha^{s0}$	0.003	−0.001
Preference for government bond	$\alpha^{B0}$	−0.001	0.0002
Euro Area preference for international bonds	$\alpha_{\mathrm{EA}}^{bw0}$	0.015	0.008
Member State preference for international bonds	$\alpha^{bw0}$	−0.006	−0.006
Preference for imports from RoW	$s_{\mathrm{RoW}}^{M}$	0.526	0.473
Preference for imports from REA	$s_{\mathrm{REA}}^{M}$	0.474	0.527
Import share in consumption	$s^{M,C}$	0.264	0.216
Import share in investment	$s^{M,I}$	0.365	0.288
Import share in government expenditure	$s^{M,G}$	0.098	0.097
Import share in export	$s^{M,X}$	0.313	0.312
***Production***			
Cobb-Douglas labour share	α	0.65	0.65
Depreciation of capital stock	δ	0.014	0.014
Elasticity of substitution between differentiated goods	$\sigma^{Y}$	8.04	5.53
Depreciation of public capital stock	$\delta^{G}$	0.014	0.012
Share of commodities in total output	$s^{\mathrm{oil}}$	0.015	0.015
Linear capacity utilisation adj. costs	$\gamma^{u,1}$	0.017	0.015
Linear labour hoarding adj. cost	$\gamma^{\mathrm{fn},1}$	−0.439	−0.440
Excise duty	τ^Oil^	0.30	0.30
***Fiscal policy***			
Consumption tax	$\tau^{c}$	0.20	0.20
Corporate profit tax	$\tau^{k}$	0.30	0.30
Labour tax	$\tau^{N}$	0.389	0.352
Deficit target (in % of GDP)	${\mathrm{DEF}}_{\;}^{T}$	0.500	0.500
Debt target (in % of GDP)	${\bar{B}}_{\;}^{g}$	62.80	60.02
***Steady-state shares (in % of GDP)***			
Private investment share	I/Y	0.173	0.203
Government consumption share	G/Y	0.188	0.185
Government investment share	IG/Y	0.022	0.035
Transfers share	T/Y	0.165	0.137
Trade balance share	TB/Y	0.004	−0.011
***EA Monetary policy***			
Interest rate persistence	$\rho^{i}$	0.898	0.898
Response to inflation	$\eta^{i\pi }$	1.390	1.390
Response to output gap	$\eta^{\mathrm{iy}}$	0.085	0.085
**Others**			
Member State size (in % of world GDP)	size	4.63	1.85
Rest of Euro Area size (in % of world GDP)	${\mathrm{size}}_{\mathrm{REA}}$	12.23	15.01
Rest of the World size (in % of world GDP)	${\mathrm{size}}_{\mathrm{RoW}}$	83.14	83.14

**Table B2:** Estimated exogenous shock processes in REA and RoW.

		Prior distribution		Posterior distribution	
		Distr	Mean		
			St.dev.	DE	ES
***Autocorrelation of forcing variables***					
REA subjective discount factor	$\rho^{c}$	Beta	0.5	0.73	0.74
			0.2	(0.70, 0.86)	(0.73, 0.84)
REA mark-up shock	$\rho^{\mathrm{MUY}}$	Beta	0.5	0.21	0.18
			0.2	(0.05, 0.027)	(0.10, 0.35)
RoW subjective discount factor	$\rho^{c}$	Beta	0.5	0.88	0.87
			0.2	(0.74, 0.9)	(0.77, 0.89)
RoW mark-up shock	$\rho^{\mathrm{MUY}}$	Beta	0.5	0.86	0.91
			0.2	(0.82, 0.93)	(0.84, 0.94)
Bilateral export price MS vs RoW	$\rho^{X}$	Beta	0.5	0.97	0.84
			0.2	(0.95, 0.98)	(0.82, 0.95)
Bilateral export price REA vs RoW	$\rho^{X}$	Beta	0.5	0.43	0.45
			0.2	(0.29, 0.86)	(0.14, 0.81)
RoW import demand	$\rho^{M}$	Beta	0.5	0.98	0.98
			0.2	(0.98, 0.99)	(0.97, 0.99)
Bilateral import demand MS vs RoW	$\rho^{M}$	Beta	0.5	0.95	0.95
			0.2	(0.91, 0.97)	(0.87, 0.95)
***Standard deviation (%) of innovations to forcing variables***					
REA subjective discount factor	$\varepsilon^{c}$	Gamma	1	2.00	1.43
			0.4	(1.03, 2.53)	(0.74, 1.88)
REA mark-up shock	$\varepsilon^{\mathrm{MUY}}$	Gamma	1	0.17	0.19
			0.4	(0.14, 0.20)	(0.15, 0.21)
RoW subjective discount factor	$\varepsilon^{c}$	Gamma	1	0.48	0.52
			0.4	(0.39, 1.15)	(0.35, 0.91)
RoW mark-up shock	$\varepsilon^{\mathrm{MUY}}$	Gamma	1	0.11	0.02
			0.4	(0.05, 0.14)	(0.02, 0.13)
Bilateral export price MS vs RoW	$\varepsilon^{X}$	Gamma	1	2.47	3.16
			0.4	(1.86, 2.61)	(2.50, 3.24)
RoW import demand	$\varepsilon^{M}$	Gamma	1	2.98	3.03
			0.4	(2.69, 3.76)	(2.85, 3.80)
Bilateral import demand MS vs RoW	$\varepsilon^{M,k}$	Gamma	2	5.12	4.58
			0.8	(4.14, 5.21)	(3.84, 4.92)
RoW monetary policy	$\varepsilon^{i}$	Gamma	1	0.07	0.06
			0.4	(0.06, 0.08)	(0.06, 0.08)

Note: Cols. (1)-(2) list model innovations. Cols. (3)-(4) indicate the prior distribution function. Identical priors are assumed for REA and RoW parameters. Cols. (5)-(8) show the mode and the (10% and 90%) HPD intervals of the posterior distributions.

**Table B3:** Model fit for DE baseline model.

	Data		Model		r^2^
Variable	Std(%)	Corr (x,GDP)	Std(%)	Corr (x,GDP)	1y-ahead
***DE***					
GDP growth (GY)	0.84	1.00	1.04	1.00	0.49
Consumption growth (GC)	0.57	0.25	1.12	0.43	−3.35
std(GC)/std(GY)	0.68		1.08		
Investment growth (GI)	2.14	0.78	2.35	0.60	0.39
std(GI)/std(GY)	2.55		2.26		
GDP deflator	0.33	−0.20	0.54	−0.39	0.69
Δ Trade balance to GDP	0.73	0.33	0.79	0.34	0.82
***REA***					
GDP growth (GY)	0.55	1.00	0.60	1.00	0.73
GDP deflator	0.30	0.24	0.32	0.10	0.89
Δ Trade balance to GDP	0.44	−0.22	0.61	−0.16	0.88
***RoW***					
GDP growth (GY)	0.62	1.00	0.35	1.00	0.96
GDP deflator	0.35	0.78	0.25	0.48	0.86
Δ Trade balance to GDP	0.08	0.07	0.11	−0.04	0.88

Note: The r^2^ is reported for the absolute nominal trade balance.

**Table B4:** Model fit for ES baseline model.

	Data		Model		r^2^
Variable	Std(%)	Corr (x,GDP)	Std(%)	Corr (x,GDP)	1y-ahead
***ES***					
GDP growth (GY)	0.68	1.00	0.62	1.00	0.80
Consumption growth (GC)	0.85	0.82	0.97	0.64	0.86
std(GC)/std(GY)	1.26		1.56		
Investment growth (GI)	2.46	0.67	2.45	0.39	0.53
std(GI)/std(GY)	3.62		3.95		
GDP deflator	0.48	0.58	0.41	−0.10	0.91
Δ Trade balance to GDP	0.66	−0.44	0.72	−0.12	0.92
***REA***					
GDP growth (GY)	0.61	1.00	0.67	1.00	0.79
GDP deflator	0.22	0.02	0.26	0.06	0.92
Δ Trade balance to GDP	0.42	0.09	0.58	−0.01	0.69
***RoW***					
GDP growth (GY)	0.62	1.00	0.32	1.00	0.96
GDP deflator	0.36	0.78	0.25	0.59	0.65
Δ Trade balance to GDP	0.08	0.08	0.11	0.02	0.82

Note: The r^2^ is reported for the absolute nominal trade balance.

**Fig. B.3.1:**
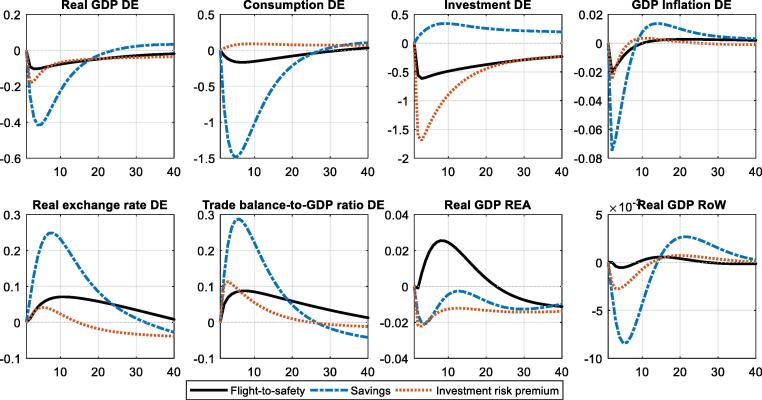
IRFs for key demand shocks in Germany (DE). Note: Simulations use the DE baseline model. Shock sizes correspond to one estimated standard deviation. An increase in the real exchange rate corresponds to a real effective depreciation. Units on the x-axis are quarters, units on the y-axis are percentage-point deviations from the steady state (trade balance) and per cent (%) deviations from steady state (all other variables), respectively.

**Fig. B.3.2:**
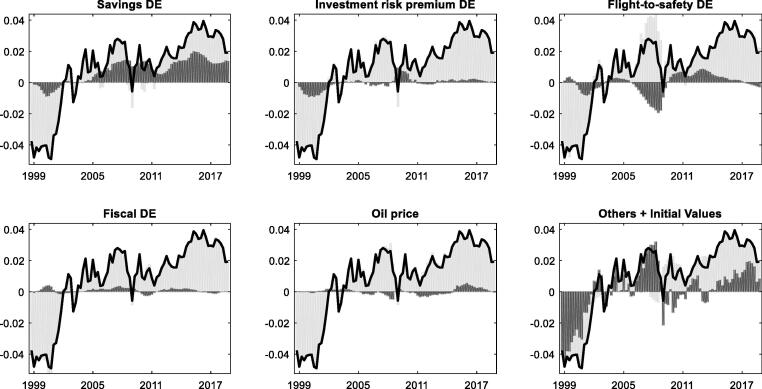
Selected shock contributions to TBY in Germany (DE). Note: Units on the x-axis are years and units on the y-axis measure the trade balance (as a share of GDP) relative to its sample mean, where 0.01 corresponds to 1% of GDP. Black bars show the contributions of the respective shock group.

**Fig. B.3.3:**
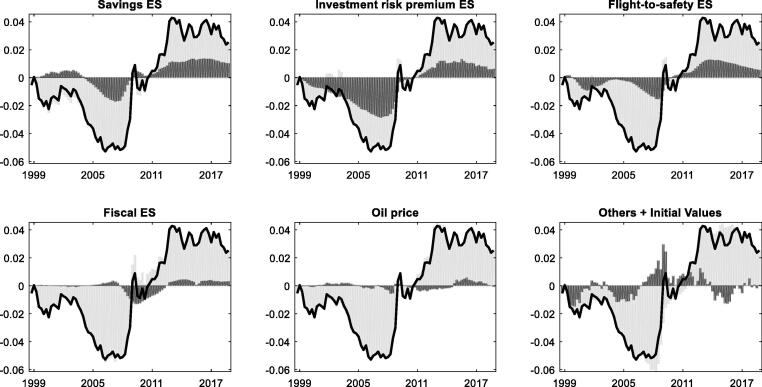
Selected shock contributions to TBY in Spain (ES). Note: Units on the x-axis are years and units on the y-axis measure the trade balance (as a share of GDP) relative to its sample mean, where 0.01 corresponds to 1% of GDP. Black bars show the contributions of shock groups.

**Fig. B.4.1:**
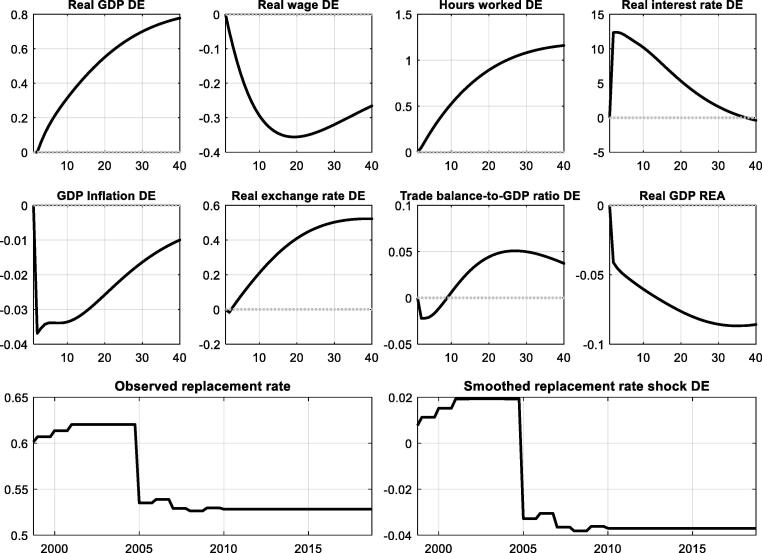
Replacement rate shocks in Germany (DE). Note: Dynamic effects of a negative shock to the replacement rate (shock size of 1 pp.). Simulations use the DE baseline model. An increase in the real exchange rate corresponds to a real effective depreciation. Units on the x-xis are quarters, units on the y-axis are percentage-point deviations from the steady state (trade balance) and percent (%) deviations from steady state (all other variables), respectively. The real interest rate is expressed in annualised basis points. The lower two panels depict the observed replacement rate and the corresponding estimated shock process in the model.

**Fig. B.5.1:**
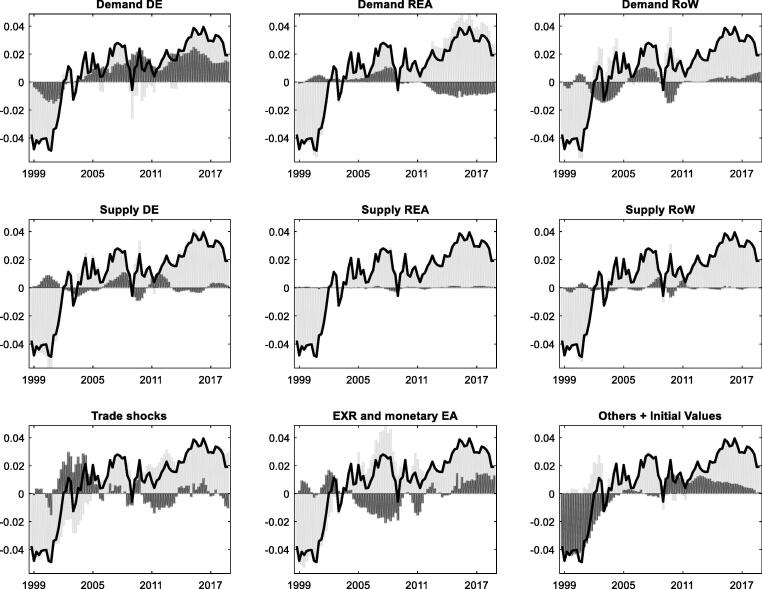
Shock decomposition of the German trade balance-to-GDP ratio. Note: Units on the x-axis are years and units on the y-axis measure the trade balance as share of GDP relative to its sample mean, where 0.01 corresponds to 1% of GDP.

**Fig. B.5.2:**
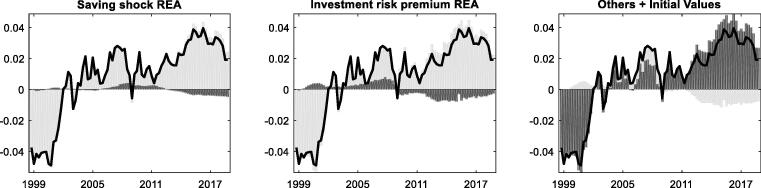
Disaggregation of REA demand shocks in decomposition of DE TBY. Note: Units on the x-axis are years and units on the y-axis measure the trade balance as share of GDP relative to its sample mean, where 0.01 corresponds to 1% of GDP.

**Fig. B.5.3:**
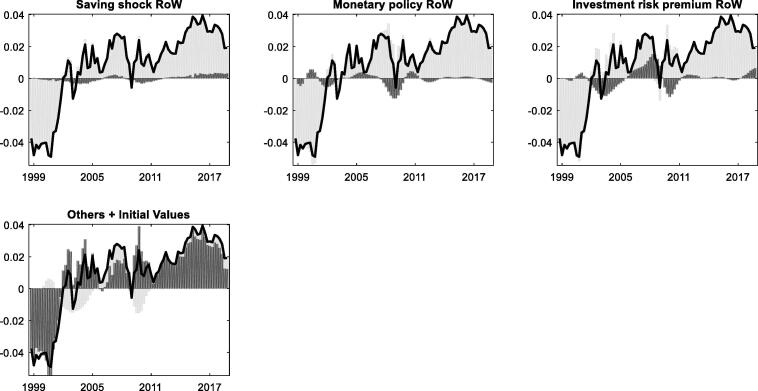
Disaggregation of RoW demand shocks in decomposition of DE TBY. Note: Units on the x-axis are years and units on the y-axis measure the trade balance as share of GDP relative to its sample mean, where 0.01 corresponds to 1% of GDP.

**Table C1:** Observed time series.

Member state (DE and ES)	Euro area	RoW
Real GDP	Real GDP	Real GDP
TFP trend	GDP trend	GDP trend
Worked hours	Effective exchange rate (nom.)	Oil price
Nominal wage share to GDP	Nominal interest rate	Nominal interest rate
Import share to GDP	Nominal trade balance to GDP	Population
Export share to GDP	Nominal exports	
Nominal government transfers to GDP	Population	
Nominal government debt to GDP		
Nominal government interest payments to GDP		
Nominal government consumption to GDP		
Nominal total investment to GDP		
Nominal private consumption to GDP		
Nominal exports to GDP		
Nominal imports to GDP		
GDP deflator		
Government consumption to GDP deflator		
Government investment to GDP deflator		
Private consumption to GDP deflator		
Total investment to GDP deflator		
Nominal import to GDP deflator		
Nominal exports to GDP deflator		
Nominal trade balance to GDP		
Active population rate		
Population		

Note: We observe EA aggregate variables and compute model-consistent REA observation equations. We observe the first quarter of the capital stock and the net international investment position as starting points.

## Appendices

### A Model description

This Appendix provides the full equilibrium conditions of the household and firm optimization problems.

The Ricardian household problem leads to the following first-order conditions (FOC):

The FOC w.r.t. savers' consumption produces:

$$\Theta_{\mathrm{kt}}{\left(C_{\mathrm{kt}}^{s}-hC_{\mathrm{kt}-1}^{s}\right)}^{-\theta }=\lambda_{\mathrm{kt}}^{s}$$

where $\lambda_{\mathrm{kt}}^{s}$ is the Lagrange multiplier on the budget constraint.

The FOC w.r.t. domestic risk-free bond:

$$\beta E_{t}\left[\frac{\lambda_{\mathrm{kt}+1}^{s}}{\lambda_{\mathrm{kt}}^{s}}\frac{1+i_{\mathrm{kt}}^{\mathrm{rf}}}{1+\pi_{\mathrm{kt}+1}^{C,vat}}\right]=1$$

The FOC w.r.t. domestic government bonds:

$$\beta E_{t}\left[\frac{\lambda_{\mathrm{kt}+1}^{s}}{\lambda_{\mathrm{kt}}^{s}}\frac{1+i_{\mathrm{kt}}^{g}-\varepsilon_{\mathrm{kt}}^{B}-\alpha_{k}^{b0}}{1+\pi_{\mathrm{kt}+1}^{C,vat}}\right]=1$$

with $\pi_{\mathrm{kt}}^{C,vat}$ the consumption deflator inflation rate and $\varepsilon_{\mathrm{kt}}^{B}$ the risk-premium on government bonds.

The FOC w.r.t. domestic stocks:

$$\beta E_{t}\left[\frac{\lambda_{\mathrm{kt}+1}^{s}}{\lambda_{\mathrm{kt}}^{s}}\frac{\left(1+i_{\mathrm{kt}+1}^{s}\right)-\varepsilon_{\mathrm{kt}}^{S}-\alpha_{k}^{s0}}{1+\pi_{\mathrm{kt}+1}^{C,vat}}\right]=1$$

where $\varepsilon_{\mathrm{kt}}^{S}$ the risk premium on stocks. The above optimality conditions are similar to a textbook Euler equation, but incorporate asset-specific risk premia that depend on an exogenous shock $\varepsilon_{\mathrm{kt}}^{Q}$ as well as the size of the asset holdings as a share of GDP; see Vitek (2017) for a similar formulation. Taking into account the Euler equation for the risk-free bond and approximating simplifies the FOCs to the familiar expressions:

$$i_{\mathrm{kt}}^{g}=i_{kt}^{rf}+\alpha_{k}^{b0}+\varepsilon_{kt}^{B}=i_{\mathrm{kt}}^{\mathrm{rf}}+{\mathrm{rprem}}_{\mathrm{kt}}^{g}$$

$$E_{t}[i_{kt+1}^{s}]=i_{kt}^{rf}+\alpha_{k}^{s0}+\varepsilon_{kt}^{S}=i_{\mathrm{kt}}^{\mathrm{rf}}+{\mathrm{rprem}}_{\mathrm{kt}}^{s}$$

In the equations above, ${\mathrm{rprem}}_{\mathrm{kt}}^{g}$ is the risk premium on domestic government bonds, and ${\mathrm{rprem}}_{\mathrm{kt}}^{s}$ is a risk premium on domestic equity (shares).^20^

Given the monetary union setting, we assume that an uncovered interest rate parity condition links the interest rate of the individual EMU region to the EA interest rate (set by the central bank):

$$\left(1+i_{\mathrm{kt}}^{\mathrm{rf}}\right)=\left(1+i_{\mathrm{EA},t}\right)-\left(\alpha_{k}^{bw1}\frac{e_{\mathrm{RoW},EA,t}B_{\mathrm{kt}}^{bw}}{P_{\mathrm{kt}}^{Y}Y_{\mathrm{kt}}}\right)+\varepsilon_{\mathrm{kt}}^{\mathrm{FQ}}$$

where $\alpha_{k}^{bw1}\frac{e_{\mathrm{RoW},EA,t}B_{\mathrm{kt}}^{bw}}{P_{\mathrm{kt}}^{Y}Y_{\mathrm{kt}}}$ captures a debt-dependent country risk premium on net foreign asset (NFA) holdings to ensure the long-run stability of foreign debt (see, e.g., Schmitt-Grohe and Uribe, 2003). Following Smets and Wouters (2007), we also introduce an additional risk premium shock, $\varepsilon_{\mathrm{kt}}^{\mathrm{FQ}}$ (‘flight-to-safety’), which creates a wedge between the EA interest rate, $i_{\mathrm{EA},t}$, and the return on domestic risk-free assets, $i_{\mathrm{kt}}^{\mathrm{rf}}$. Since a positive shock increases the required return on domestic assets and the cost of capital, it reduces current consumption and investment simultaneously and helps explaining the co-movement of consumption and investment.

The instantaneous utility functions for liquidity-constrained households, $u^{c}\left(∙\right)$, is defined as:

$$u_{\mathrm{jkt}}^{c}\left(C_{\mathrm{jkt}}^{c},N_{\mathrm{jkt}}^{c}\right)=\frac{1}{1-\theta_{k}}{\left(C_{\mathrm{jkt}}^{c}-h_{k}C_{\mathrm{kt}-1}^{c}\right)}^{1-\theta_{k}}-{\left(C_{\mathrm{kt}}^{\;}\right)}^{1-\theta_{k}}\frac{\omega_{k}^{N}\varepsilon_{\mathrm{kt}}^{U}}{1+\theta_{k}^{N}}{\left(N_{\mathrm{jkt}}^{c}\right)}^{1+\theta_{k}^{N}}$$

with $C_{\mathrm{kt}}^{c}=\int_{\omega^{s}}^{1}C_{\mathrm{jkt}}^{c}dj$.

***Total output***

Perfectly competitive firms produce total output, $O_{\mathrm{kt}}$, by combining value added, $Y_{\mathrm{kt}}$, with energy input, ${\mathrm{Oil}}_{\mathrm{kt}}$, using the following CES production function:

$$O_{\mathrm{kt}}={\left[{\left(1-s_{k}^{\mathrm{Oil}}\right)}^{\frac{1}{\sigma_{k}^{o}}}{\left(Y_{\mathrm{kt}}\right)}^{\frac{\sigma_{k}^{o}-1}{\sigma_{k}^{o}}}+{\left(s_{k}^{\mathrm{Oil}}\right)}^{\frac{1}{\sigma_{k}^{o}}}{\left({\mathrm{Oil}}_{\mathrm{kt}}\right)}^{\frac{\sigma_{k}^{o}-1}{\sigma_{k}^{o}}}\right]}^{\frac{\sigma_{k}^{o}}{\sigma_{k}^{o}-1}},$$

where $s_{k}^{\mathrm{Oil}}$ is the energy input share in total output and $\sigma_{k}^{o}$ is the elasticity of substitution between both components. It follows that demand for $Y_{\mathrm{kt}}$ and ${\mathrm{Oil}}_{\mathrm{kt}}$ by total output producers is, respectively:

$$Y_{\mathrm{kt}}=\left(1-s_{k}^{\mathrm{Oil}}\right){\left(\frac{P_{\mathrm{kt}}^{Y}}{P_{\mathrm{kt}}^{O}}\right)}^{-\sigma_{k}^{o}}O_{kt},$$

$${\mathrm{Oil}}_{\mathrm{kt}}=s_{k}^{\mathrm{Oil}}{\left(\frac{P_{\mathrm{kt}}^{\mathrm{Oil}}}{P_{\mathrm{kt}}^{O}}\right)}^{-\sigma_{k}^{o}}O_{kt}$$

where $P_{\mathrm{kt}}^{Y}$ and $P_{\mathrm{kt}}^{\mathrm{Oil}}$ are price deflators associated with $Y_{\mathrm{kt}}$ and ${\mathrm{Oil}}_{\mathrm{kt}}$, respectively. Oil is imported from RoW, the oil price is given by: $P_{\mathrm{kt}}^{\mathrm{Oil}}=e_{\mathrm{RoW},EA,t}P_{\mathrm{RoW},t}^{\mathrm{Oil}}+\tau^{\mathrm{Oil}}P_{\;}^{Y0}$, where $\tau^{\mathrm{Oil}}P_{\;}^{Y0}$ is the excise duty. The price index of total output $P_{\mathrm{kt}}^{O}$ is:

$$P_{\mathrm{kt}}^{O}={\left[\left(1-s_{k}^{\mathrm{Oil}}\right){\left(P_{\mathrm{kt}}^{Y}\right)}^{1-\sigma_{k}^{o}}+s_{k}^{\mathrm{Oil}}{\left(P_{\mathrm{kt}}^{\mathrm{Oil}}\right)}^{1-\sigma_{k}^{o}}\right]}^{\frac{1}{1-\sigma_{k}^{o}}}$$

***Firms***

Each variety i is produced by a single firm using total capital, $K_{\mathrm{ikt}-1}^{\mathrm{tot}}$, and labour, $N_{\mathrm{ikt}}^{\;}$, which are combined by a Cobb-Douglas production function:

$$Y_{\mathrm{ikt}}={\left[A_{\mathrm{kt}}^{Y}\left(N_{\mathrm{ikt}}-{\mathrm{FN}}_{\mathrm{ik}}\right)\right]}^{\alpha }{\left({\mathrm{cu}}_{\mathrm{ikt}}K_{\mathrm{ikt}-1}^{\mathrm{tot}}\right)}^{1-\alpha }-A_{\mathrm{kt}}^{Y}{\mathrm{FC}}_{k},$$

where α is the steady-state labour share, $A_{\mathrm{kt}}^{Y}$ is labour-augmenting productivity common to all firms in the differentiated goods sector, and ${\mathrm{cu}}_{\mathrm{ikt}}$ and ${\mathrm{FN}}_{\mathrm{ik}}$ are the firm-specific levels of capital utilisation and labour hoarding, respectively.^21^ Total capital, $K_{\mathrm{ikt}}^{\mathrm{tot}}$, is the sum of privately installed capital, $K_{\mathrm{ikt}}$, and public capital, $K_{\mathrm{kt}}^{G}$. ${\mathrm{FC}}_{k}$ captures fixed costs in production. Economy-wide total factor productivity, ${\mathrm{TFP}}_{\mathrm{kt}},$ is:

$${\mathrm{TFP}}_{\mathrm{kt}}={\left(A_{\mathrm{kt}}^{Y}\right)}^{\alpha }$$

Since TFP is a non-stationary process, we allow for two types of shocks that are related to a non-stationary process and its autoregressive component, respectively:

$$\log\left({\bar{A}}_{\mathrm{kt}}^{Y}\right)-log\left({\bar{A}}_{\mathrm{kt}-1}^{Y}\right)=g_{\mathrm{kt}}^{\bar{\mathrm{AY}}}+\varepsilon_{\mathrm{kt}}^{\mathrm{lay}},$$

$$g_{\mathrm{kt}}^{\bar{\mathrm{AY}}}=\rho^{\bar{\mathrm{AY}}}g_{\mathrm{kt}-1}^{\bar{\mathrm{AY}}}+\left(1-\rho^{\bar{\mathrm{AY}}}\right)g^{\bar{AY0}}+\varepsilon_{\mathrm{kt}}^{\mathrm{gay}},$$

where $g_{t}^{\bar{\mathrm{AY}}}$ and $g^{\bar{AY0}}$ are the time-varying growth and the long-run growth of technology, respectively. $\varepsilon_{\mathrm{kt}}^{\mathrm{lay}}$ and $\varepsilon_{\mathrm{kt}}^{\mathrm{gay}}$ are permanent technology level and growth rate shock, respectively.

The monopolistically competitive producers maximise the real value of the firm, $V_{i\mathrm{kt}}$, equal to the discounted stream of future dividends $d_{i\mathrm{kt}}$, $V_{i\mathrm{kt}}=d_{i\mathrm{kt}}+E_{t}\left[{\mathrm{sdf}}_{\mathrm{kt}+1}V_{i\mathrm{kt}+1}\right]$, with the stochastic discount factor:

$${\mathrm{sdf}}_{\mathrm{kt}}=E_{t}\left[\frac{1+i_{kt+1}^{s}}{1+\pi_{kt+1}^{y}}\right]\approx E_{t}\left[\frac{1+i_{kt}^{rf}+rprem_{kt}^{s}}{1+\pi_{kt+1}^{y}}\right],$$

which depends directly on the investment risk premium, ${\mathrm{rprem}}_{\mathrm{kt}}^{s}$. The dividends are defined as:

$$d_{\mathrm{ikt}}=\left(1-\tau_{\;}^{K}\right)\left(\frac{P_{\mathrm{ikt}}^{Y}}{P_{\mathrm{kt}}^{Y}}Y_{\mathrm{ikt}}-\frac{W_{\mathrm{kt}}}{P_{\mathrm{kt}}^{Y}}N_{\mathrm{ikt}}\right)+\tau_{\;}^{K}\delta_{k}\frac{P_{\mathrm{kt}}^{I}}{P_{\mathrm{kt}}^{Y}}K_{ikt-1}-\frac{P_{\mathrm{kt}}^{I}}{P_{\mathrm{kt}}^{Y}}I_{\mathrm{ikt}}-{\mathrm{adj}}_{\mathrm{ikt}},$$

where $I_{\mathrm{ikt}}$ is physical investment, $P_{\mathrm{kt}}^{I}$ is the investment price, $\tau_{k}^{K}$ is the corporate tax, and $\delta_{k}$ is the capital depreciation rate.

Following (Rotemberg, 1982), adjustment costs, ${\mathrm{adj}}_{\mathrm{ikt}}$, are associated with the output price, $P_{\mathrm{ikt}}^{Y}$, labour input, $N_{\mathrm{ikt}}$, capacity utilisation, ${\mathrm{cu}}_{\mathrm{ikt}}$, investment, $I_{\mathrm{ikt}}$, and labour hoarding, ${\mathrm{FN}}_{\mathrm{ikt}}$:

$${\mathrm{adj}}_{\mathrm{ikt}}=adj\left(P_{\mathrm{ikt}}^{Y}\right)+adj\left(N_{\mathrm{ikt}}\right)+adj\left({\mathrm{cu}}_{\mathrm{ikt}}\right)+adj\left(I_{\mathrm{ikt}}\right)+adj\left({\mathrm{FN}}_{\mathrm{ikt}}\right)$$

where:

$${\mathrm{adj}}_{\mathrm{ikt}}^{\mathrm{PY}}=\frac{\gamma^{p}}{2}Y_{\mathrm{kt}}{\left(\frac{P_{\mathrm{ikt}}^{Y}}{P_{\mathrm{ikt}-1}^{Y}}-1\right)}^{2}$$

$${\mathrm{adj}}_{\mathrm{ikt}}^{N}=\frac{\gamma^{n}}{2}Y_{\mathrm{kt}}{\left(\frac{N_{\mathrm{ikt}}-{\mathrm{FN}}_{\mathrm{ikt}}}{N_{\mathrm{ikt}-1}-{\mathrm{FN}}_{\mathrm{ikt}-1}}-1\right)}^{2}$$

$${\mathrm{adj}}_{\mathrm{ikt}}^{\mathrm{cu}}=\frac{P_{\mathrm{kt}}^{I}}{P_{\mathrm{kt}}^{Y}}K_{\mathrm{ikt}-1}\left(\gamma^{u,1}\left({\mathrm{cu}}_{\mathrm{ikt}}-1\right)+\frac{\gamma^{u,2}}{2}{\left({\mathrm{cu}}_{\mathrm{ikt}}-1\right)}^{2}\right)$$

$${\mathrm{adj}}_{\mathrm{ikt}}^{I}=\frac{P_{\mathrm{kt}}^{I}}{P_{\mathrm{kt}}^{Y}}\left[{\frac{\gamma^{I,1}}{2}K}_{\mathrm{kt}-1}{\left(\frac{I_{\mathrm{ikt}}}{K_{\mathrm{kt}-1}}-\delta \right)}^{2}+\frac{\gamma^{I,2}}{2}\frac{{\left(I_{\mathrm{ikt}}-I_{\mathrm{ikt}-1}\right)}^{2}}{K_{\mathrm{kt}-1}}\right]$$

$${\mathrm{adj}}_{\mathrm{ikt}}^{\mathrm{FN}}=Y_{\mathrm{kt}}\left[\gamma^{\mathrm{fn},1}{\left(\frac{{\mathrm{FN}}_{\mathrm{ikt}}}{{\mathrm{Actr}}_{\mathrm{kt}}{\mathrm{Pop}}_{\mathrm{kt}}}-\overset{¯}{\mathrm{FN}}\right)}^{\;}+\frac{\gamma^{\mathrm{fn},2}}{2}{\left(\frac{{\mathrm{FN}}_{\mathrm{ikt}}}{{\mathrm{Actr}}_{\mathrm{kt}}{\mathrm{Pop}}_{\mathrm{kt}}}-\overset{¯}{\mathrm{FN}}\right)}^{2}\right]$$

in which the γ parameters capture the degree of adjustment costs, and ${\mathrm{Actr}}_{\mathrm{kt}}{\mathrm{Pop}}_{\mathrm{kt}}$ is the active labour force. The maximization is subject to the production function, the standard capital accumulation equation, $K_{\mathrm{ikt}}=\left(1-\delta \right)K_{\mathrm{ikt}-1}+I_{\mathrm{ikt}}$, and the usual demand condition that inversely links the demand for variety *i* goods to the price of the variety:

$$Y_{\mathrm{ikt}}={\left(\frac{P_{\mathrm{ikt}}^{Y}}{P_{\mathrm{kt}}^{Y}}\right)}^{-\sigma^{y}}Y_{kt}$$

The usual equality between the marginal product of labour and labour cost holds, with a wedge driven by the labour adjustment costs:

$$\mu_{\mathrm{kt}}^{y}\alpha \frac{Y_{\mathrm{kt}}}{N_{\mathrm{kt}}-{\mathrm{FN}}_{\mathrm{kt}}}-{\mathrm{adj}}_{\mathrm{ikt}}^{N}=\left(1-\tau_{k}^{K}\right)\frac{W_{\mathrm{kt}}}{P_{\mathrm{kt}}^{Y}}$$

with $\mu_{\mathrm{kt}}^{y}$ being inversely related to the price markup. The capital optimality condition reflects the usual dynamic trade-off faced by the firm:

$$E_{t}\left[\frac{1+\pi_{kt+1}^{y}}{1+i_{kt+1}^{s}}\frac{P_{kt+1}^{I}/P_{kt+1}^{Y}}{P_{kt}^{I}/P_{kt}^{Y}}\left(\mu_{kt+1}^{y}\left(1-\alpha \right)\frac{P_{kt+1}^{Y}Y_{ikt+1}}{P_{kt+1}^{I}K_{ikt}^{tot}}+\tau_{\;}^{K}\delta -\frac{adj_{kt}^{cu}}{K_{ikt}}+\left(1-\delta \right)\;Q_{kt+1}\right)\right]=Q_{\mathrm{kt}}$$

where $Q_{\mathrm{kt}}$ has the usual Tobin's *Q* interpretation.

The FOC w.r.t. investment implies that Tobin's *Q* varies due to investment adjustment costs:

$$Q_{\mathrm{kt}}=1+{\mathrm{adj}}_{\mathrm{ikt}}^{I}.$$

Firms adjust their capacity utilisation and labour hoarding depending on market conditions in line with the respective optimality conditions:

$$\frac{\mu_{\mathrm{kt}}^{y}}{P_{\mathrm{kt}}^{I}/P_{\mathrm{kt}}^{Y}}\left(1-\alpha \right)\frac{Y_{\mathrm{kt}}}{{\mathrm{cu}}_{\mathrm{kt}}}={\mathrm{adj}}_{\mathrm{ikt}}^{\mathrm{cu}}$$

$$\mu_{\mathrm{kt}}^{y}\alpha \frac{Y_{\mathrm{kt}}}{N_{\mathrm{kt}}-{\mathrm{FN}}_{\mathrm{kt}}}={\mathrm{adj}}_{\mathrm{ikt}}^{\mathrm{FN}}$$

Finally, the FOC w.r.t. the price of differentiated varieties of goods pins down the price markup:

$$\frac{\sigma^{y}}{\left(\sigma^{y}-1\right)}\mu_{\mathrm{kt}}^{y}=\left(1-\tau_{\;}^{K}\right)+\frac{{\mathrm{adj}}_{\mathrm{ikt}}^{\mathrm{PY}}}{\left(\sigma^{y}-1\right)}+\varepsilon_{\mathrm{kt}}^{\mathrm{MUY}},$$

with $\varepsilon_{\mathrm{kt}}^{\mathrm{MUY}}$being the markup shock. The latter equation, combined with the FOC w.r.t. labour and symmetry in pricing behaviour, implies the Phillips curve of the familiar form:

$$\gamma_{\mathrm{kt}}^{y}\sigma_{k}^{Y}=\left(1-\tau_{\;}^{K}\right)\left(\sigma_{k}^{Y}-1\right)+\gamma_{k}^{P}\sigma_{k}^{Y}\frac{P_{\mathrm{kt}}^{Y}}{P_{\mathrm{kt}-1}^{Y}}\left[E_{t}\pi_{\mathrm{kt}}^{Y}-\bar{\pi }\right]-\gamma_{k}^{P}\sigma_{k}^{Y}E_{t}\left[\frac{1+\pi_{\mathrm{kt}+1}^{Y}}{1+i_{\mathrm{kt}+1}^{s}}\frac{P_{\mathrm{kt}+1}^{Y}}{P_{\mathrm{kt}}^{Y}}\frac{Y_{\mathrm{kt}+1}^{\;}}{Y_{\mathrm{kt}}^{\;}}\left(\pi_{\mathrm{kt}+1}^{Y}-\bar{\pi }\right)\right]+\sigma_{k}^{Y}\varepsilon_{\mathrm{kt}}^{\mathrm{MUY}},$$

where $\varepsilon_{\mathrm{kt}}^{\mathrm{MUY}}$ is the inverse of the price markup shock.

***Labour markets***

The optimality condition for the equilibrium wage is given by:

$${\left(\mu_{k}^{w}\frac{U_{\mathrm{kt}}^{N}}{\lambda_{\mathrm{kt}}^{\;}}\frac{P_{\mathrm{kt}}^{C,vat}}{P_{\mathrm{kt}}^{Y}}\right)}^{1-\gamma_{k}^{\mathrm{wr}}}{\left(\left(1-\tau_{k}^{N}\right)\frac{W_{\mathrm{kt}-1}}{P_{\mathrm{kt}-1}^{C,vat}}\right)}^{\gamma_{k}^{\mathrm{wr}}}=\left(1-\tau_{k}^{N}\right)\frac{W_{\mathrm{kt}}}{P_{\mathrm{kt}}^{C,vat}}{+\gamma }_{k}^{w}\left(\frac{W_{\mathrm{kt}}}{W_{\mathrm{kt}-1}}-1-\left(1-{\mathrm{sf}}_{k}^{w}\right)\left(\pi_{\mathrm{kt}-1}^{y}-\bar{\pi }\right)-\pi^{w}\right)\frac{W_{\mathrm{kt}}}{W_{\mathrm{kt}-1}}\frac{W_{\mathrm{kt}}}{P_{\mathrm{kt}}^{Y}}-\gamma_{k}^{w}E_{t}\left[{\overset{\;}{\beta }}_{\mathrm{kt}}\frac{\lambda_{\mathrm{kt}+1}}{\lambda_{\mathrm{kt}}}\frac{N_{\mathrm{kt}+1}}{N_{\mathrm{kt}}}\frac{P_{\mathrm{kt}}^{C,vat}}{P_{\mathrm{kt}+1}^{C,vat}}\left(\frac{W_{\mathrm{kt}+1}}{W_{\mathrm{kt}}}-1-\left(1-{\mathrm{sf}}_{k}^{w}\right)\left(\pi_{\mathrm{kt}}^{y}-\bar{\pi }\right)-\pi^{w}\right)\frac{W_{\mathrm{kt}+1}}{W_{\mathrm{kt}}}\frac{W_{\mathrm{kt}}}{P_{\mathrm{kt}}^{Y}}\right]+\varepsilon_{\mathrm{kt}}^{U}\frac{W_{\mathrm{kt}}}{P_{\mathrm{kt}}^{Y}}$$

where $\varepsilon_{k}^{w}$ is the wage mark-up, $\gamma_{k}^{\mathrm{wr}}$ is the degree of real wage rigidity, $\gamma_{k}^{w}$ is the degree of nominal wage rigidity, and ${\mathrm{sf}}_{k}^{w}$ is the degree of forward-lookingness in the labour supply equation. $U_{\mathrm{kt}}^{N}$ is the marginal disutility of labour:

$$U_{\mathrm{kt}}^{N}=\omega_{k}^{N}{\left(C_{\mathrm{kt}}\right)}^{1-\theta_{k}}{\left(N_{\mathrm{kt}}^{\;}\right)}^{{-\theta }_{k}^{N}}$$

In the case of Germany, the real wage term on the left-hand side accounts for unemployment benefits, i.e. we modify $\left(1-\tau_{k}^{N}\right)$ to $\left(1-\tau_{k}^{N}-b_{\mathrm{kt}-1}^{U}\right)$, where $b_{\mathrm{kt}-1}^{U}$ is the replacement rate. Similalry, the term $\left(1-\tau_{k}^{N}\right)$becomes $\left(1-\tau_{k}^{N}-b_{\mathrm{kt}}^{U}\right)$ on the right-hand side.

***REA and RoW demand functions***

From profit maximisation we obtain the demand for domestic and foreign goods:

$$Y_{\mathrm{kt}}^{}={\left(A_{\mathrm{kt}}^{p}\right)}^{\sigma_{k}^{c}-1}\left(1-s_{kt}^{M}\right){\left(\frac{P_{\mathrm{kt}}^{Y}}{P_{\mathrm{kt}}^{C}}\right)}^{-\sigma_{k}^{c}}C_{kt}$$

$$M_{\mathrm{kt}}^{}={\left(A_{\mathrm{kt}}^{p}\right)}^{\sigma_{k}^{c}-1}s_{kt}^{M}{\left(\frac{P_{\mathrm{kt}}^{M}}{P_{\mathrm{kt}}^{C}}\right)}^{-\sigma_{k}^{c}}C_{kt}$$

where the consumer price deflator, $P_{\mathrm{kt}}^{C}$, is given by:

$$P_{\mathrm{kt}}^{C}={\left(A_{\mathrm{kt}}^{p}\right)}^{-1}{\left[\left(1-s_{kt}^{M}\right){\left(P_{\mathrm{kt}}^{Y}\right)}^{1-\sigma_{k}^{c}}+s_{kt}^{M}{\left(P_{\mathrm{kt}}^{M}\right)}^{1-\sigma_{k}^{c}}\right]}^{\frac{1}{1-\sigma_{k}^{c}}}$$

***RoW monetary policy***

Monetary policy in RoW follows a Taylor-type rule similar to the EA one:

$$i_{\mathrm{RoW},t}-\bar{i}=\rho_{\mathrm{RoW}}^{i}\left(i_{\mathrm{RoW},t-1}-\bar{i}\right)+\left(1-\rho_{\mathrm{RoW}}^{i}\right)\left[\eta_{\mathrm{RoW}}^{i\pi }0.25\left(\pi_{\mathrm{RoW},t}^{c,vat,QA}-{\bar{\pi }}_{\mathrm{RoW}}^{c,vat,QA}\right)+\eta_{\mathrm{RoW}}^{\mathrm{iy}}\left(log\left(0.25\sum_{r=1}^{4}Y_{\mathrm{RoW},t-r}\right)-log\left(0.25\sum_{r=1}^{4}Y_{\mathrm{RoW},t-r}^{\mathrm{pot}}\right)\right)\right]+\varepsilon_{\mathrm{RoW},t}^{i}.$$

***RoW oil endowment***

The RoW supplies inelastically its unstorable exogenous endowment:

$${\mathrm{Oil}}_{\mathrm{RoW},t}=\frac{size_{k}}{size_{RoW}}Oil_{kt}+\frac{size_{REA}}{size_{RoW}}Oil_{REA,t},$$

the price of which, $P_{\mathrm{RoW},t}^{\mathrm{Oil}}$ is set (exogenously) in RoW currency.

### B Additional results

#### B.1 Calibrated parameters, steady state ratios and estimated exogenous processes

See Table B1 and Table B2.

#### B.2 Theoretical moments and model fit

Table B3 and Table B4 compare sample and model-implied moments for a subset of statistics to evaluate the capability of the benchmark model to fit the data. Specifically, we report the volatilities of real GDP, consumption, investment, the trade balance-to-GDP ratio, and the GDP deflator as well as the cross-correlation of GDP with its main components. We use first differences for the trade balance-to-GDP ratio and quarter-on-quarter growth rates for all other variables. Most of the correlations between GDP growth and its components are well captured. Our estimated model for DE tends to overestimate the volatility of real variables, while the ES model matches the data moments reasonably well. The relative magnitudes are preserved. In particular, the high volatility of investment is in line with the data. These theoretical moments show that the estimated structural models successfully replicate key business cycles features in DE and ES.

The last column reports the r^2^ of the 1-year ahead forecast. We define the r^2^ as 1 minus the ratio between the k-step ahead forecast error and the deviation of the observed time series from the model-implied steady state. This definition implies that r^2^ has an upper bound at 1 and is unbounded from below. In the perfect case where the model generates no forecast error, the r^2^ is equal to one, whereas in the case where the volatility of the forecast error is larger than the volatility of the observed time series, the r^2^ is negative. The positive r^2^ values indicate that the model forecast errors are not very large. However, the model-implied volatility of consumption growth in DE is higher than its empirical counterpart.

#### B.3 Details on the estimated effects of private demand shocks, discretionary fiscal policy, and oil price shocks

This section provides additional details on the contributions of private demand shocks (B.3.1), fiscal policy (B.3.2), and oil price shocks (B.3.3) to the dynamics of the TB.

##### B.3.1 Private domestic demand shocks

The model includes three private domestic demand shocks: Shocks to preferences for risk-free bonds (“flight-to-safety” shocks), shocks to the discount factor (“savings shocks”), and shocks to the investment risk premium. While our main results are robust to the exclusion of one of these shocks, the model fit and identification critieria support the specification with all three shocks.

Fig. B.3.1 illustrates that each of the three private domestic demand shocks triggers distinct dynamics in the economy, which allows to identify all three shocks separately. Adverse demand shocks improve the TB relative to GDP (TBY) in all three cases, but the transmission differs markedly. The private savings shocks (dashed blue) reduces consumption and lowers domestic and foreign GDP and inflation. Yet, the shock crowds in private investment. By contrast, the investment risk premium shock (dotted red) dampens investment. The fall in aggregate demand lowers GDP and inflation, but the model-predicted crowding-in of consumption is at odds with the observed co-movement of domestic demand components. The flight-to-safety shock (solid black) reduces aggregate demand (consumption *and* investment), inflation, and GDP, which suggests that this shock can account for salient features of the recent recessions, especially in the case of ES.. The boom-bust cycle in ES has featured a simultaneous strong decline in consumption and investment, together with a reversal of TB from high deficit to surplus (Fig. B.3.3).

##### B.3.2 Fiscal shocks

Fiscal shocks play only a small role for DE TBY dynamics (Fig. B.3.2). In ES, the initial fiscal stimulus during the Global financial crisis counteracted the private demand contraction, which shows up as negative contribution to TBY. Starting around 2012, however, the model estimation identifies reductions in government spending and investment (“austerity”) as contributions to the post-crisis TB reversal in ES (Fig. B.3.3).

##### B.3.3 Oil price shocks

The model includes an oil price shock as exogenous supply-side change in the price of oil (in RoW currency). Because EA and its MS are oil importers, any change in the oil price will affect the TBY, i.e. a lower (higher) oil price will reduce (increase) the import bill and improve (deteriorate) the TB. Giovannini et al. (2019) show that oil prices (and a broader bundle of commodities) were indeed relevant drivers of the TBY in the EA and the US after the Great Recession.^22^ We find that oil price hikes deteriorated the TBY in 2008 and 2011/12, whereas the oil price decline in 2015–17 led to an improvement in DE and ES external positions relative to the long-term average. The estimated dynamics are similar for both DE and ES, i.e. they do not explain differences in the overall TBY pattern between the two economies.

#### B.4 Hartz reforms - estimated effects of replacement rate shocks

In 2003–2005, the DE government implemented a far-reaching labour market deregulation, the so-called ‘Hartz’ reforms, which aimed at better job matching and reduced the length and generosity of unemployment benefits (the benefit replacement rate fell permanently from 62% to 53% on average in 2004–2005). The discussion and model implementation here closely follows Kollmann et al. (2015). In particular, we capture the structural change by observing the benefit replacement rate (see lower panel in Fig. B.4.1) in this model. Unemployment benefits (paid to unemployed workers in the labour force) enter the budget constraints of the households and the government. A random-walk shock to the replacement rate captures the observed (permanent) structural change. Appendix A above provides the formal details.

Fig. B.4.1 displays dynamic responses to the shock to the benefit replacement rate. A cut in the replacement rate stimulates DE labour supply, which lowers the real wage rate and increases employment in DE persistently. Initially, GDP increases faster than the TB, but after around 10 quarters the competitiveness gain improves the TBY persistently. The aggregate supply shock is thus consistent with the increasing TB surplus after 2005.

#### B.5 Model extension - adding capital to the REA and RoW model blocks

As a further robustness check, we have extended the REA and RoW model blocks by including capital and investment decisions to assess whether the simplified structure of the benchmark model may bias results against a stronger impact of foreign drivers by omitting “pull factors” of net capital exports.^23^ Fig. B.5.1 shows that domestic demand factors remain the main driver of the DE TBY ratio.

The total contribution of REA and RoW demand shocks in Fig. B.5.1 is similar to Fig. 3. The extended model rather decomposes the contribution of foreign aggregate demand shocks to DE TBY further into foreign household savings versus investment risk premium shocks (Fig. B.5.2, Fig. B.5.3).

### C Data transformations

We use quarterly and annual data for the period 1999q1 to 2018q4. Data for EMU countries (DE and ES) and the Euro Area aggregate (EA19) are taken from Eurostat (in particular, from the European System of National Accounts ESA2010). Bilateral trade flows are based on trade shares from the GTAP trade matrices for trade in goods and services. The rest of the world (RoW) data are annual data and are constructed using IMF International Financial Statistics (IFS) and World Economic Outlook (WEO) databases.

Series for GDP and prices in the RoW start in 1999 and are constructed on the basis of data for the following 58 countries: Albania, Algeria, Argentina, Armenia, Australia, Azerbaijan, Belarus, Brazil, Bulgaria, Canada, Chile, China, Colombia, Croatia, Czech Republic, Denmark, Egypt, Georgia, Hong Kong, Hungary, Iceland, India, Indonesia, Iran, Israel, Japan, Jordan, Korea, Lebanon, Libya, FYR Macedonia, Malaysia, Mexico, Moldova, Montenegro, Morocco, New Zealand, Nigeria, Norway, Philippines, Poland, Romania, Russia, Saudi Arabia, Serbia, Singapore, South Africa, Sweden, Switzerland, Syria, Taiwan, Thailand, Tunisia, Turkey, Ukraine, United Arab Emirates, United Kingdom, and Venezuela.

When not available, quarterly-frequency data are obtained by interpolating annual data. We seasonally adjust the following time series: nominal public investments (for EA19, DE, and ES), nominal social benefits other than transfers in kind (for EA19, DE, and ES), government interest expenditure (for EA19, DE, and ES), compensation of employees (for DE, and ES), and general government net lending (for ES).

Table C.1 lists the observed time series. We apply logarithmic transformations to all observables, with the exception of the trade balance-to-GDP ratio, the oil price, and nominal interest rates. GDP deflators and relative prices of demand components are computed as the ratios of the current-price value to the chain-indexed volume series. In DE, we additionally observe the historical average unemployment benefit ratio (constructed as the ratio of unemployment benefits to the wage rate).

We make a few transformations to the raw investment series. In particular, we compute the deflator of public investments based on annual data and then obtain its quarterly-frequency counterpart through interpolation. This series together with nominal public investments is then used to compute real quarterly public investments. In order to assure consistency between nominal GDP and the sum of the nominal components of aggregate demand, we impute the change in inventories to the series of investments.

## Footnotes

1 The models build on the Global Multi-country (GM) model of the European Commission (Albonico et al., 2019).

2 Unless stated differently, all exogenous random variables in the model follow independent autoregressive processes.

3 As in Benigno (2009) and Ratto et al. (2009), we assume that only the RoW bond is traded internationally.

4 Internationally traded bonds are subject to a risk premium that depends on the average net foreign asset (NFA) position relative to GDP.

5 Since both households face the same labour demand schedule, each household works the same number of hours as the average of the economy. It follows that the individual union's choice variable is a common nominal wage rate for both types of households. See Appendix A for additional details.

6 Productivity is a non-stationary stochastic process subject to trend and level shocks (see Appendix A).

7 According to Burnside and Eichenbaum (1996), firms prefer not to lay off workers when the demand is temporarily low because firing workers may be more costly than hoarding them. Additionally, the inclusion of labour hoarding helps matching the observed co-movement between output and working hours.

8 Since we allow for a non-zero trade balance in the steady state, we include an international transfer, $\mathrm{IT}R_{k}$, calibrated to satisfy zero NFA in equilibrium.

9 Appendix B.5 shows that our main results remain unaffected when we extend the model by capital formation and multi-input production functions in the REA and the RoW. For clarity, we thus choose the simpler approach outlined here as the benchmark.

10 In contrast, since RoW is an oil exporter, the budget constraint for the representative household is:$P_{\mathrm{RoWt}}^{Y}Y_{\mathrm{RoWt}}+P_{\mathrm{RoWlt}}^{\mathrm{Oil}}{\mathrm{Oil}}_{\mathrm{lt}}=P_{\mathrm{RoWt}}^{C}C_{\mathrm{RoW},t}+{\mathrm{TB}}_{\mathrm{RoWt}}$,where $P_{\mathrm{RoWt}}^{Y}$ and $Y_{\mathrm{RoWt}}$ are price and volume of RoW final good output, $P_{\mathrm{RoWlt}}^{\mathrm{Oil}}$ and ${\mathrm{Oil}}_{\mathrm{RoWlt}}$ are price and volume of oil exports to country *l*=(EMU regions), and ${\mathrm{TB}}_{\mathrm{RoWt}}$ is the trade balance. For simplicity, oil is an unstorable exogenous endowment of RoW and is supplied inelastically. The price of oil, $P_{\mathrm{RoWt}}^{\mathrm{Oil}}$, is determined in RoW currency.

11 The endogenous risk premium ensures long-run stability of the NFA position (see, e.g., Schmitt-Grohe and Uribe, 2003).

12 We use the DYNARE software (Adjemian et al., 2011). The estimated model includes 39 exogenous shocks, as it appears that many shocks are needed to capture the dynamic properties of the macroeconomic and financial data (e.g., Kollmann et al., 2015). The large number of shocks is also dictated by the fact that we use a large number of observables (38) for the estimation. For details on slice sampling, see Neal (2003) and Planas et al. (2015).

13 Table B2 in Appendix B.1 provides the estimated exogenous shock processes in REA and RoW for both model versions (DE and ES).

14 We model the (“Hartz”) reforms as permanent shock to the unemployment benefit replacement rate. The estimated trade balance effects of this policy change are highly persistent. Appendix B.4 provides additional details.

15 For example, a difference in the log data density of 1 implies that a model is preferred by a log Bayes factor of 1. See e.g., Kass and Raftery (1995).

16 In the approach in Subsection 6.1, the original shocks remain in the price or wage equations, i.e. the coefficients on the initial shocks equal one.

17 In practice, the size of $\hat{Y}$ in a multi-region macro model is very large. We do not include ASDs in equations that are pure definitions (e.g., of growth rates) or accounting identities. Given the size of our model and the data set, we focus discussion on entries of $\hat{Y}$ in the main behavioural equations.

18 Theoretically, it would be interesting to extend the ASD approach to all shocks entering the model and even allow for more than one ASD at once. Given the large number of shocks and possible configurations, this remains virtually impossible, however.

19 See also Devereux and Sutherland (2011) on the role of leverage constraints for the international transmission of shocks.
